# Targeting Sialidase to PD1 Enhances T cell Function and Tumor Control

**DOI:** 10.1021/acscentsci.5c00510

**Published:** 2025-07-04

**Authors:** Brett M. Garabedian, Eleanor E. Bashian, Xiaoshuang Wang, Andrew J. Thompson, James C. Paulson

**Affiliations:** † Department of Immunology and Microbiology, 4356 The Scripps Research Institute, La Jolla, California 92037, United States; ‡ Department of Molecular and Cellular Biology, 4356 The Scripps Research Institute, La Jolla, California 92037, United States

## Abstract

Immune therapies targeting the PD1 axis have transformed outcomes in cancer treatment by enhancing T cell-mediated immune responses. However, many tumors evade immune clearance through orthogonal escape mechanisms. Excessive production of immunosuppressive sialic acid-containing glycans (sialoglycans) can impair immune surveillance by recruiting inhibitory Siglecs to the immune synapse where, like PD1, they act as checkpoints for cell activation. Sialic acids can also impact T cell activation by dampening the ligation of the costimulatory receptor CD28 with its ligands. This polypharmacology implicates sialoglycans as a linchpin of tumor immunity that can be targeted to further improve outcomes of PD1 therapies. In this work we conjugated sialidase to anti-PD1 (αPD1-S) to selectively degrade sialic acids on immune cells expressing PD1. Glycan profiling confirmed targeted desialylation, and functional assays demonstrated enhancements to T cell activation and cytotoxic capacity. In a melanoma model, αPD1-S promoted inflammatory macrophage polarization and reduced T cell exhaustion, collectively restricting melanoma growth beyond anti-PD1 (αPD1) alone. By simultaneously blocking PD1 and degrading sialoglycans, αPD1-S provides a novel strategy to enhance T cell-mediated immune responses and improve tumor control in refractory cancers.

## Graphical abstract

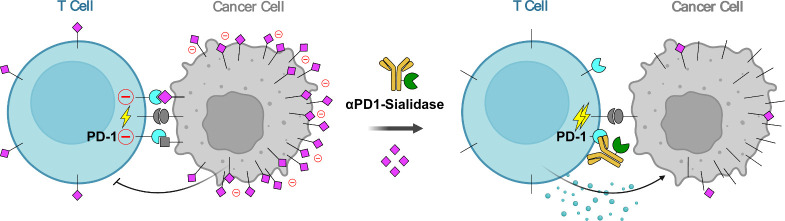

## Background

The tumor microenvironment (TME) promotes rapid growth of transformed cells while suppressing resident immune cell effector functions. Hypersialylation contributes to these effects and is characterized by aberrantly high levels of sialic acid-containing glycans (sialoglycans) that serve as signatures of “self” to suppress immune function. Sialoglycans recruit inhibitory sialic acid-binding immunoglobulin-like lectin (Siglec) receptors on leukocytes that recognize sialic acids as ligands.^1,2^ While Siglec-sialoglycan engagement suppresses autoimmunity in healthy cells,^3^ cancer cells co-opt this mechanism to evade the immune response. Indeed, Siglec interactions with sialoglycans on tumor cells reduce Natural Killer (NK)-mediated cell killing^4,5^ and regulate tumor-associated macrophage (TAM) polarization to a reparative M2 phenotype,^6–10^ while Siglec-9 is highly expressed on tumor-infiltrating T cells and suppresses effector function in the tumor microenvironment.^11,12^

The importance of sialic acids in T cell activation was recognized long ago when it was found that enzymatic removal of sialic acids from T cells (*in cis*) or from antigen-presenting cells (APCs) (*in trans*) enhances antigen mediated T cell activation, referred to as the “neuraminidase effect”.^13–22^ Mechanistically, this enhancement may result in part from our finding that sialic acids on T cells or APCs reduce binding of the T cell costimulatory receptor, CD28 to its B7 ligands CD80/86 on APCs, dampening this critical coactivator and diminishing T cell activation.^23^ Functionally, the “neuraminidase effect” is also relevant to findings from several groups showing T cell-mediated clearance of B16F10 tumors is enhanced by reducing tumor cell sialic acids via intratumoral injection^24^ of a sialyltransferase inhibitor,^25^ or by genetic knockdown of a CMP-sialic transporter.^26^ Together these data demonstrate removal of sialoglycans from the TME enhances T cell tumor immunity, providing precedent for additional targeted therapies that deploy the neuraminidase effect.

An emerging modality to reduce sialoglycans in the TME includes targeting sialidase to tumor cells or tumor-infiltrating lymphocytes.^27–31^ To achieve this, researchers fused sialidase from *Vibrio cholerae*, *Salmonella typhimurium*, *Homo sapiens* or *Bifidobacterium infantis* to antibodies specific for various tumor antigens. In the case of human epidermal growth factor receptor-2 (HER2), targeted conjugates selectively removed sialic acids from HER2+ cells with a potent antitumor response in murine model of breast cancer.^28^ The authors recapitulated delayed tumor growth rates in MC38 tumors lacking UDP-N-acetylglucosamine 2-epimerase (GNE knockout, KO) essential to sialic acid biosynthesis, correlating these delays in tumor growth with decreased tumor sialic acids levels.^10^ Critically, depletion of CD8^+^ T cells in mice harboring MC38-GNE-KO tumors rescued tumor growth and abrogated the efficacy of HER2-targeted sialidases, highlighting a fundamental but undefined role for sialic acids that mediate CD8^+^ T cell tumor immunity. The mechanism of tumor restriction was also shown to depend on Siglec-E (functional paralog of human Siglec-9) on myeloid cells and was synergistic with checkpoint therapy.

Taken together, we reasoned direct targeting of sialidase to T cells could enhance antitumor immune responses. Accordingly, we fused sialidase to antibodies targeting the clinically validated checkpoint receptor, Programmed Cell Death-1 (PD1) highly expressed on tumoral T cells. Because signaling through CD28 is required for the efficacy of PD1 blockade,^32–37^ we sought to enhance αPD1 therapy by removing sialic acids on T cells (*in cis*) and/or APCs (*in trans*), thereby promoting engagement of CD28/B7 at the immune synapse. The resulting αPD1-sialidase conjugate is designed to reinvigorate hypofunctional T cells by two synergistic mechanisms: (i) classically inhibiting the PD1-L1/L2 immune axis^38,39^ and (ii) targeting sialidase to PD1+ cells in the TME to locally deploy the “neuraminidase effect” and potentiate antitumor immune responses (Figure 1).

**Figure 1:**
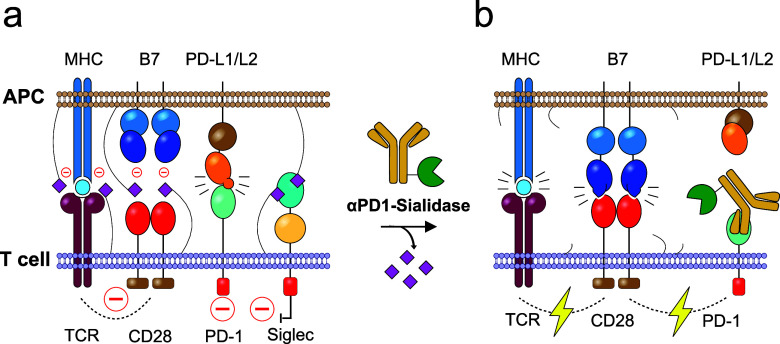
Enhancing antitumor T cell immunity with an αPD1-sialidase conjugate. (a) Sialoglycans (purple diamonds) on T cells and antigen-presenting cells (APCs) hinder immune synapse formation, reduce T cell receptor (TCR)-CD28 costimulation, and recruit inhibitory Siglecs, leading to impaired T cell activation. (b) The αPD1-sialidase conjugate binds to exhausted T cells, blocking PD1-mediated inhibitory signaling while simultaneously removing sialoglycans from T cells and APCs. This prevents inhibitory Siglec recruitment at the immune synapse and enhances T cell activation.

## Results

### Conjugation of Sialidase to αPD1

As a preferred method for conjugation of sialidase to an anti-murine PD1 (αPD1) we employed the bacterial transpeptidase Sortase A (SrtA) mutant “8M” that enzymatically couples proteins containing a C-terminal SrtA consensus “LPXTG” motif to proteins containing a N-terminal polyglycine motif.^40^ For production of αPD1, the gene fragments of the variable heavy and light regions of the antimurine PD1 clone J43 ^41^ or the antihuman PD1 clone 409A11 (Keytruda)^42^ were cloned onto a human IgG4 Fc with the C-terminal LPETG sortase tag on the heavy chain C-terminus (Figure 2a, Supplementary Figure S1). We similarly generated an isotype control antibody (motavizumab) targeting respiratory syncytial virus (RSV) (Supplementary Figure S1).^28^ The IgG4 subclass was chosen for its inability to bind Fc receptors, to abrogate the potential for antibody-dependent cell-mediated cytotoxicity of host lymphocytes.^43^ Optimized coupling conditions include 30 μM αPD1 in the presence of 20 mol equiv sialidase and 5 mol equiv SrtA, added in that order. Reactions to generate αPD1-S could be done at 60 mg scale with a coupling efficiency of ≥90%, as determined by analysis of the antibody heavy chain gel mobility shift by SDS-PAGE (Figure 2b,c). The reaction was very rapid, 1 min total reaction time at 37 °C, and was followed immediately by purification using (i) protein A, (ii) Ni-NTA IMAC and (iii) size-exclusion chromatography with an isolated yield of 83%. αPD1-S was determined to have a sialidase-antibody ratio of approximately 1.5 using SDS-PAGE (Figure 2b) and intact MALDI-TOF (Figure 2c) analysis. αPD1-S were also confirmed for retention of binding to PD1 (Figure 2d) and sialidase activity (Figure 2e) using the fluorogenic sialoglycan surrogate substrate 4-methyl­umbelli­feryl-α-d-N-acetyl­neuram­inate (MUNANA).^44^

**Figure 2:**
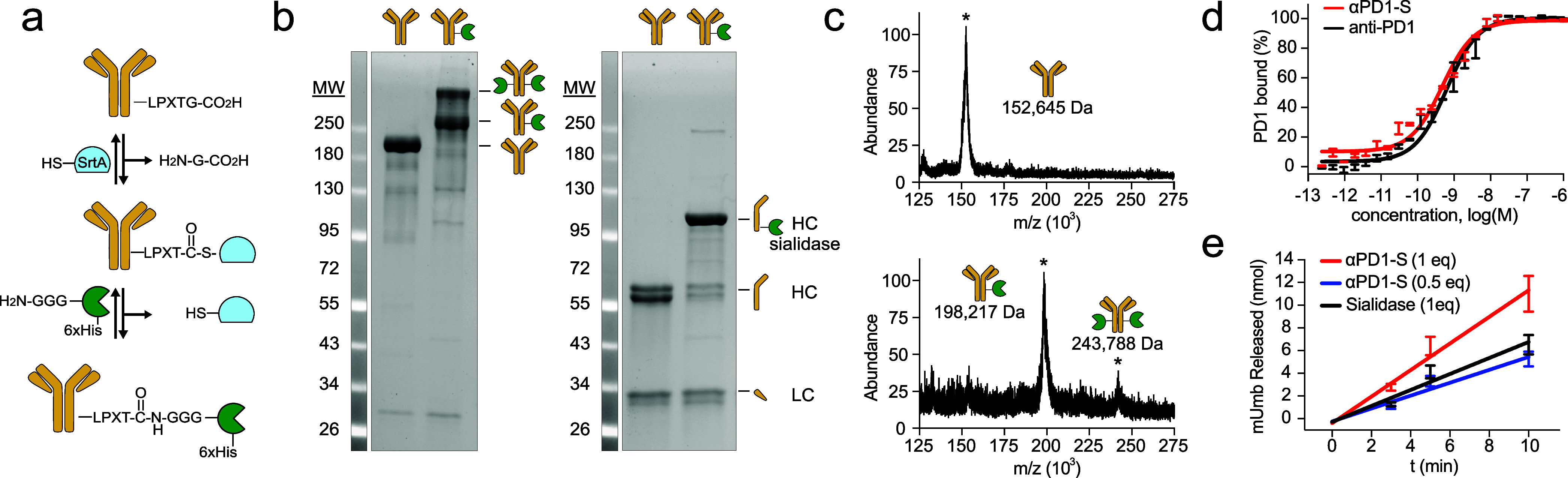
Construction of PD1-targeted sialidase (αPD1-S). (a) Anti-PD1 antibody C-terminally tagged with the “LPXTG” consensus motif was activated via thioesterification by Sortase A (SrtA) and reacted with N-terminal polyglycine-tagged sialidase to afford αPD1-S. (b) Analysis of purified αPD1-S by nonreducing (left) and reducing (right) SDS-PAGE indicating site-selective conjugation of ST sialidase to the anti-PD1 heavy chain (ratio ∼ 1.5). (c) Intact protein analysis by MALDI-TOF MS illustrating unmodified anti-PD1 (top) and αPD1-S (bottom). (d) PD1 binding analysis by ELISA, indicating αPD1-S retain affinity to PD1 following conjugation by SrtA. (e) *In vitro* activity assay of αPD1-S and ST sialidase, indicating αPD1-S retains sialidase activity following conjugation by SrtA (*n* = 3, average shown, fit to linear regression).

To mitigate potential off-target desialylation that are anticipated for long-term *in vivo*, experiments,^28^ we also developed an αPD1-S with reduced sialidase activity. To this end, we performed alanine scanning of the *S. typhimurium* sialidase active site, and identified a point mutant R309A that exhibited approximately 500-fold less active than wild-type ST sialidase (Supplementary Figure S2a,b). Accordingly, we developed an αPD1-S containing the R309A variant as described above, which we refer to as αPD1-S*. We also developed an antibody isotype sialidase (IgG4-S*) to account for sialidase targeting effects mediated by the IgG4 Fc component. As shown Supplementary Figure S2c-e, αPD1-S* retained robust on-target sialidase activity toward *in vitro* activated PD1+ T cells.

### αPD1-S Mediates Delivery of Sialidase to PD1+ T Cells

The αPD1-S conjugate was characterized for (i) PD1 binding activity on T cells, (ii) the impact of sialidase activity when targeted to the cell surface, and (iii) the functional ability to reverse T cell exhaustion *in vitro*, as described below. To demonstrate that αPD1-S binds to PD1 and assess the impact of targeting sialidase to PD1 on the cell surface, we compare αPD1-S for the ability to degrade sialic acids on Jurkat T cells that do not express PD1 (PD1-GFP-) with Jurkat T cells expressing human PD1-green fluorescent protein (PD1+GFP+), generously provided by Professor Enfu Hui at UCSD.^45^ In brief, Jurkat cells ± PD1-GFP were mixed in a 1:1 ratio and treated with hαPD1-S for 1 h at 37 °C (Figure 3a). Cells were then analyzed by flow cytometry after staining with lectins including: *Sambucus nigra* (SNA) specific for α2–6 linked sialoglycans; *Maackia amurensis* Lectin II (MAL-II) specific for α2–3 linked sialoglycans; or Peanut Agglutinin (PNA) recognizing asialoglycans (Galβ1–3GalNAc) revealed by removing α2–3 linked sialic acids on *O*-linked glycans. The results in Figure 3b-d show enhanced sialic acid removal from PD1+GFP+ cells by ≥1000-fold, demonstrating the ability to selectively degrade sialoglycans from PD1+ cells (Supplementary Figure S3). A similar assay was utilized to characterize antimurine αPD1-S using the OT-I mouse model. CD8 T cells from OT-I mice possess a transgenic TCR that recognizes chicken egg ovalbumin-derived oligopeptide “SIINFEKL” loaded on major histocompatibility complex I (MHC-I). Graded expression of PD1 on OT-I cells was achieved by stimulating OT-I splenocytes with increasing SIINFEKL concentrations. PD1-lo, PD1-medium, and PD1-hi cells were mixed in equal parts and treated with αPD1-S before lectin staining and flow cytometry analysis. The results show that greater desialylation was achieved with increasing expression of PD1 (Supplementary Figures S4 and S5).

**Figure 3:**
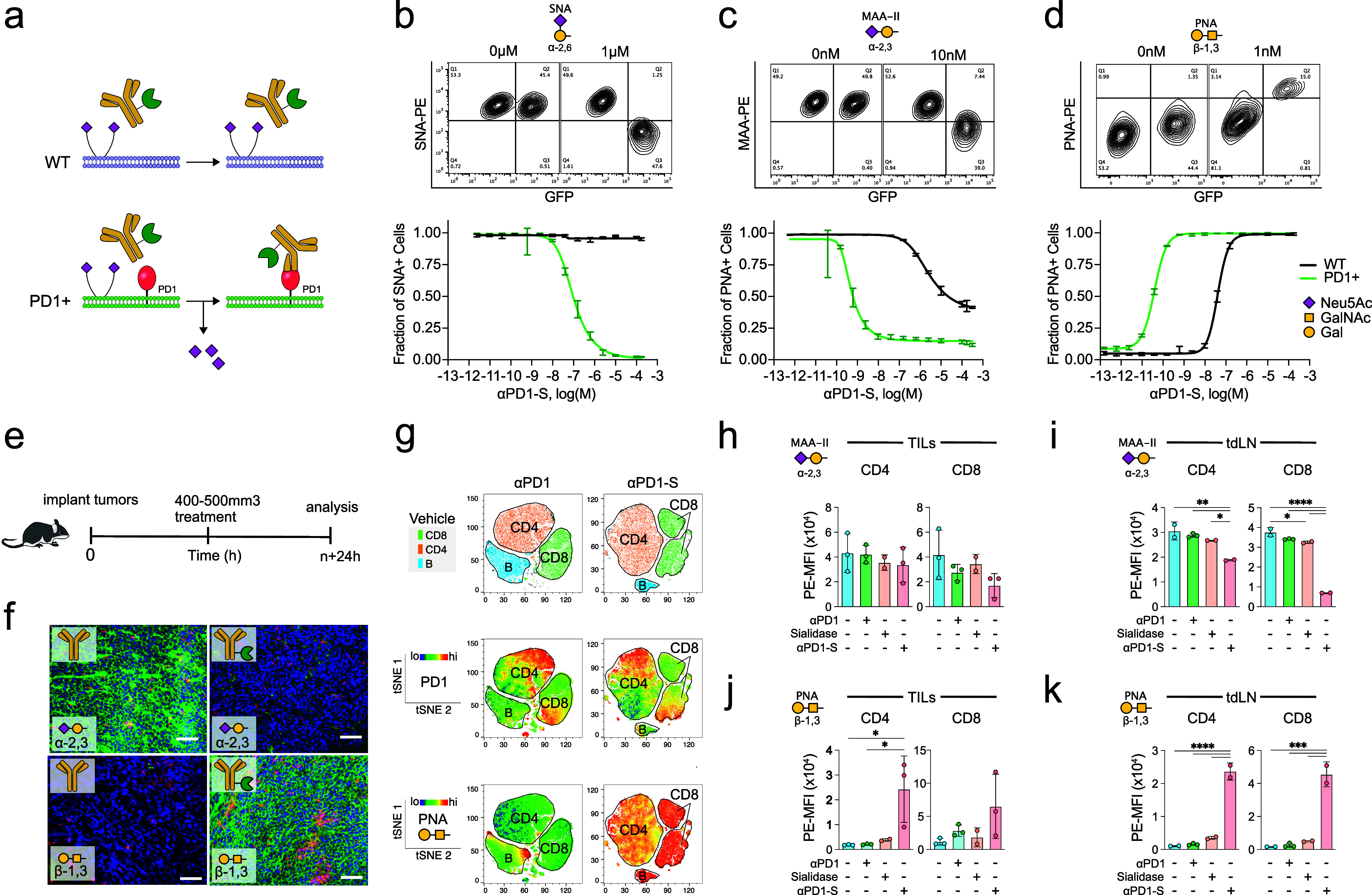
αPD1-S selectively degrades sialoglycans from PD1+ T cells. (a) Wild type (WT) and PD1­(+)­GFP­(+) Jurkat T cells were mixed in equal parts and treated with αPD1-S. (b–d) Representative contour plots (top) demonstrating reduced detection of sialoglycans by SNA (b), MAA (c) and PNA (d) on PD1­(+)­GFP­(+) cells following treatment with αPD1-S at the indicated concentration (bottom). (e) Mice bearing 500 mm^3^ CT26 tumors were treated with a single intraperitoneal injection (1 nmol in 100 μL) of anti-PD1 or αPD1-S. Tumors and lymphoid tissues are resected 24 h after treatment and analyzed by immunofluorescence imaging (f) or flow cytometry (g–k) for sialic acid abundance as determined by lectins. (f) Immunofluorescence imaging of CT26 tumor cross sections following treatment with anti-PD1 or αPD1-S. Colors indicate CD3 cells (red); lectin indicated (green); nuclear stain (DAPI; blue). Scale bar = 100 μm. (g, top) Analysis of tumor-draining lymphocytes from mice treated with anti-PD1 or αPD1-S using t-distributed stochastic neighbor embedding (t-SNE) analysis of high dimensional flow cytometry data, including markers for CD8 (CD3, CD8) and CD4 (CD3, CD4) T cells and B cells (CD19). (g, middle) PD1 expression heatmap (blue = low, red = high) among tumor-draining lymphocytes indicating PD1 enrichment on CD8 T cells, CD4 T cells and B cells. (g, bottom) PNA heatmap of tumor-draining lymphocytes indicating selective sialic removal by αPD1-S *in vivo*. (h–k) Quantitation of lectin-stained TILs and tumor-draining lymphocytes (tdLN) indicating sialic acid removal by αPD1-S. Error represented as standard deviation *p < 0.05, ***p < 0.001, ****p < 0.0001 by one-way ANOVA followed by Tukey’s multiple comparisons test.

To evaluate αPD1-S targeting *in vivo*, tumor-bearing mice were treated with a single dose of vehicle, αPD1, αPD1-S, or sialidase only (Figure 3e). Tumors and tumor-draining lymph nodes (tdLN) were resected after 24 h and analyzed by immunofluorescence microscopy (Figure 3f) and flow cytometry (Figure 3g), respectively. Tumor cross sections stained with sialic acid-specific lectin probes reveal on-target sialic acid degradation from tumor-infiltrating CD8 T cells by αPD1-S as well as off-target degradation from local tumor cells, potentially due to the mobility of αPD1-S targeted lymphocytes within the tumor microenvironment.^46^ Analysis of lymphocytes from tdLN showed that CD4 and CD8 T cell and B cell populations all express moderate levels of PD1 regardless of treatment, and that αPD1-S removes sialic acids from all populations as detected by increased staining by PNA (Figure 3g). Further analysis of CD4 and CD8 T cells from tumor infiltrating lymphocytes (TILs) and tdLN shows no loss of sialic acids with nontargeted sialidase detected with PNA or MAA, equivalent to those treated with αPD1 only (Figure 3h-k). Notably, loss of sialic acids was evident for both CD4 and CD8 T cells, particularly in tdLN, as detected by increased staining with PNA and decreased staining with MAA (Figure 3h-k). Notably, despite higher PD1 expression on CD4 T cells, greater desialylation was consistently observed on CD8 T cells, possibly reflecting intrinsic differences in glycosyltransferase regulation, including substantial downregulation of enzymes such as ST6Gal1 and ST3Gal1, and *de novo* synthesis of the asialo-CD45 upon CD8 T cell activation.^47–49^

### αPD1-S Drives Antigen-Specific T Cell Effector Function

To evaluate αPD1-S for its impact on T cell activation, OT-I splenocytes were stimulated *in vitro* with SIINFEKL peptide for 5 days (Figure 4a). OT-I cells stimulated under these conditions expressed high levels of PD1 and phenotypically resembled “exhausted” cells, as determined by the coexpression of markers including Lymphocyte Activation Gene 3 (LAG3), T cell immunoglobulin and mucin-domain containing-3 (TIM3), and the surrogate marker of transcription factor TCF1, indicative of T cell stemness, Ly108 ^50^ (Supplementary Figure S5). These OT-I cells were then restimulated for 24 h in the presence of the indicated treatments and subsequently analyzed by flow cytometry for sialic acid content (Figure 4b,c, Supplementary Figure S6) and the production of cytokines (Figure 4d-f). Targeting sialidase to T cells with αPD1-S resulted in greater desialylation than sialidase alone or sialidase with αPD1, as measured by the loss of binding of the sialic acid-specific lectin, MAA (Figure 4b) and an increase in the binding of PNA whose asialoligand is exposed by sialidase (Figure 4c). αPD1-S also caused significantly higher activation relative to αPD1, sialidase alone, or the combination of both as seen by the increase in TNFα+IFNγ+ and GzmB+IFNγ+ cells (Figure 4d-f). Notably, OT-I were similarly expanded and cocultured with B16 cells expressing chicken ovalbumin and green fluorescent protein (B16OVA-GFP), but no enhancements to B16OVA cell killing were observed, highlighting the need for more suitable models of T cell activation and cytolysis (Supplementary Figure S7).

**Figure 4:**
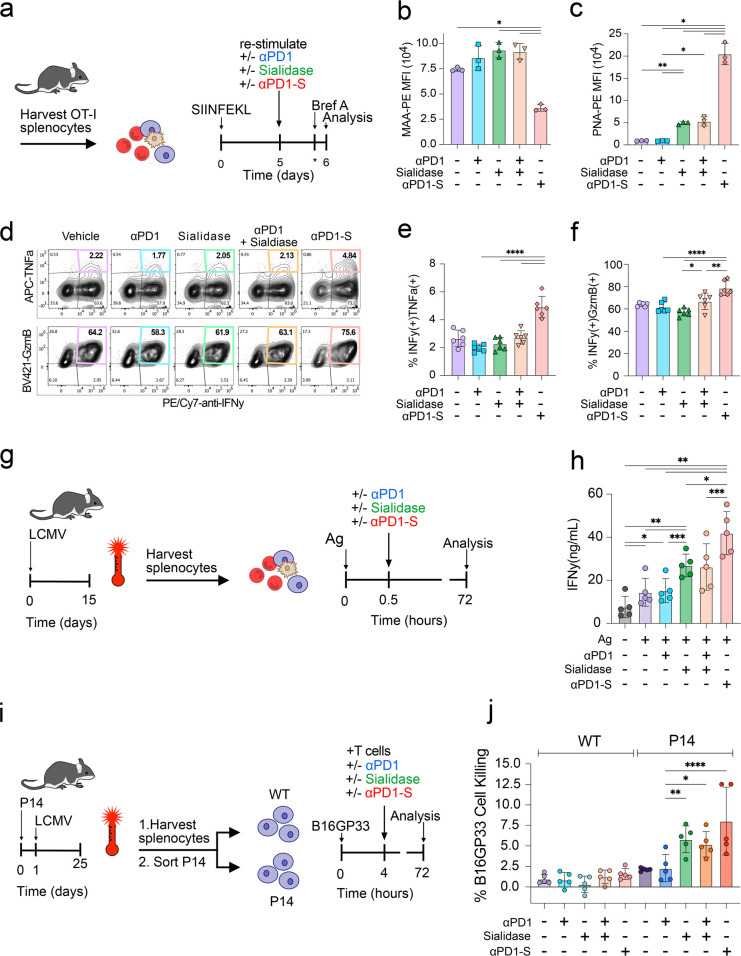
αPD1-S restores cytolytic capacity of hypofunctional T cells. (a) Splenocytes from OT-I mice were stimulated with 500 nM SIINFEKL for 5 days, followed by the indicated treatment for 1 day and subsequent analysis by flow cytometry; brefeldin A added *4 h prior to end point. (b, c) Sialic acid quantification indicating α2–3-linked sialic acid abundance (b) or Galβ1–3GalNAc abundance on O-linked glycans (c) following treatment. (d) Representative flow cytometry contour plots indicating cytokine levels (percent of total OT-I) following 24 h of the indicated treatment, quantified in (e, f). (g) Generation of hypofunctional LCMV-specific CD8 T cells from WT C57BL6/J mice. Animals were infected with LCMV clone13 and spleens harvested 15 days postinfection. Spleens were dissociated and cultured in the presence of LCMV peptide antigens (GP33, NP205, GP61, GP276, NP396; “Ag” collectively) and the indicated treatments. (h) IFNγ concentration in cell culture supernatants detected by ELISA after 72 h of treatment. (i) Generation of functionally exhausted GP33-specific P14 CD8 T cells following adoptive transfer into WT C57BL6/J mice. Mice received P14 T cells and were subsequently infected with LCMV clone13 for 25 days. Splenocytes were sorted according to Thy1.1/1.2 expression, corresponding to WT/P14, respectively. B16GP33 cells were seeded into plates and subsequently cocultured for 72 h with sorted T cells in the presence of the indicated treatments. (j) B16GP33 killing by P14 cells in the presence of the indicated treatments. Cell killing was determined by ELISA measurement of lactate dehydrogenase (LDH) in cell culture supernatants after 72 h. Error bars represent standard deviation, *p*-values were determined by matched one-way ANOVA followed by Tukey’s multiple comparisons test, where *p < 0.05, **p ≤ 0.01, ***p ≤ 0.001, ****p ≤ 0.0001.

To evaluate αPD1-S in a clinically relevant context, we turned to the well documented lymphocytic choriomeningitis virus (LCMV) infection model of CD8 T cell exhaustion.^51,52^ Briefly, naïve mice were infected with LCMV Clone 13 (generously provided by the laboratory of John Teijaro at Scripps Research), a strain that induces chronic infection,^52,53^ for 15 days. Spleens were harvested, dissociated into splenocytes, and cultured in the presence of LCMV peptide antigens (GP33, NP205, GP61, GP276, NP396; “Ag” collectively) and the indicated treatments for 72 h, and conditioned cell media were assayed for the production of IFNγ as a measure of T cell activation (Figure 4g). While αPD1 alone, sialidase alone, or a combination of both resulted in an increase in INFγ production, the αPD1-S produced significantly higher levels of IFNγ production, demonstrating a synergistic effect of PD1 blockade and sialidase.

To test whether enhanced T cell activation improved T cell killing, we utilized LCMV antigen-specific “P14” CD8^+^ T cells whose TCR recognizes the high-affinity LCMV peptide antigen GP33.^54^ To produce LCMV-specific PD1+ T cells, LCMV antigen-specific P14 cells were adoptively transferred into wild-type (WT) host mice that were then infected with LCMV Clone 13. When chronically exposed to LCMV, the adoptively transferred P14 T cells express high levels of PD1 and are functionally exhausted (Supplementary Figure S8). Host T cells (P14-) or adoptively transferred antigen specific (P14+) T cells were isolated from spleen by fluorescence-activated cell sorting and cocultured with B16 cells stably expressing the LCMV peptide antigen GP33 (B16GP33) for 72 h (Figure 4i). Target cell killing was determined by measuring lactate dehydrogenase (LDH) released into coculture supernatants, indicating cytolysis. Consistent with enhanced IFNγ production in Figure 4h, cell killing was highest in cocultures treated with αPD1-S (Figure 4j). Notably, this benefit to cell killing was only observed with the exhausted, GP33-specific P14+ cells and not with host (P14-) T cells. The results demonstrate that targeting sialidase to PD1 on hypofunctional, tumor-reactive T cells can enhance their cell killing effector phenotype.

### αPD1-S Restricts Tumor Growth in a Refractory Melanoma Model

To test the efficacy of αPD1-S *in vivo* we selected the B16OVA melanoma model which exhibits hypersialylation that suppresses effector T cells and NK cells, promotes regulatory T cells, is refractory to treatment with αPD1, and benefits from treatments that reduce sialylation.^24–26^ In particular, it has been shown that reducing sialic acids by intratumoral injection of a sialyltransferase inhibitor^24,25^ or by genetic knockdown of a CMP-sialic transporter^26^ enhances CD8 T cell-mediated control of B16OVA tumors. To evaluate αPD1-S for the ability to restrict B16OVA tumor growth, we established a threshold for adoptively transferred activated OT-I cells needed for tumor control (Supplementary Figure S9). Preliminary experiments showed that adoptive transfer of activated OT-I cells alone produced a baseline level of tumor restriction. Notably, this tumor restriction was further enhanced when mice were treated with αPD1-S* containing the R309A sialidase variant, which mitigates off-target desialylation observed with WT sialidase in Figure 3 (Supplementary Figure S2). Dosing every other day was based on a measured serum half-life of 48h and was limited to three doses due the emergence of antidrug antibodies at approximately day seven following the initial treatment (Supplementary Figure S10).

Based on these optimized protocols, OT-I splenocytes activated *ex vivo* for 5 days were adoptively transferred into four groups of 10 mice bearing B16OVA tumors measuring 100 mm^3^. Three days later, mice were treated with vehicle, αPD1, αPD1-S* or IgG4-S* every other day for a total of three treatments (Figure 5a, Supplementary Figure S1). As seen from the growth curves and Kaplan–Meier survival analysis, the αPD1-S* group exhibited strong suppression of tumor growth, which was significantly longer than αPD1 alone (Figure 5b-d). In contrast, IgG4-S* had no impact on survival. The results show the synergy achieved in tumor control by combining PD1 blockade and sialidase targeted to PD1-expressing immune cells.

**Figure 5:**
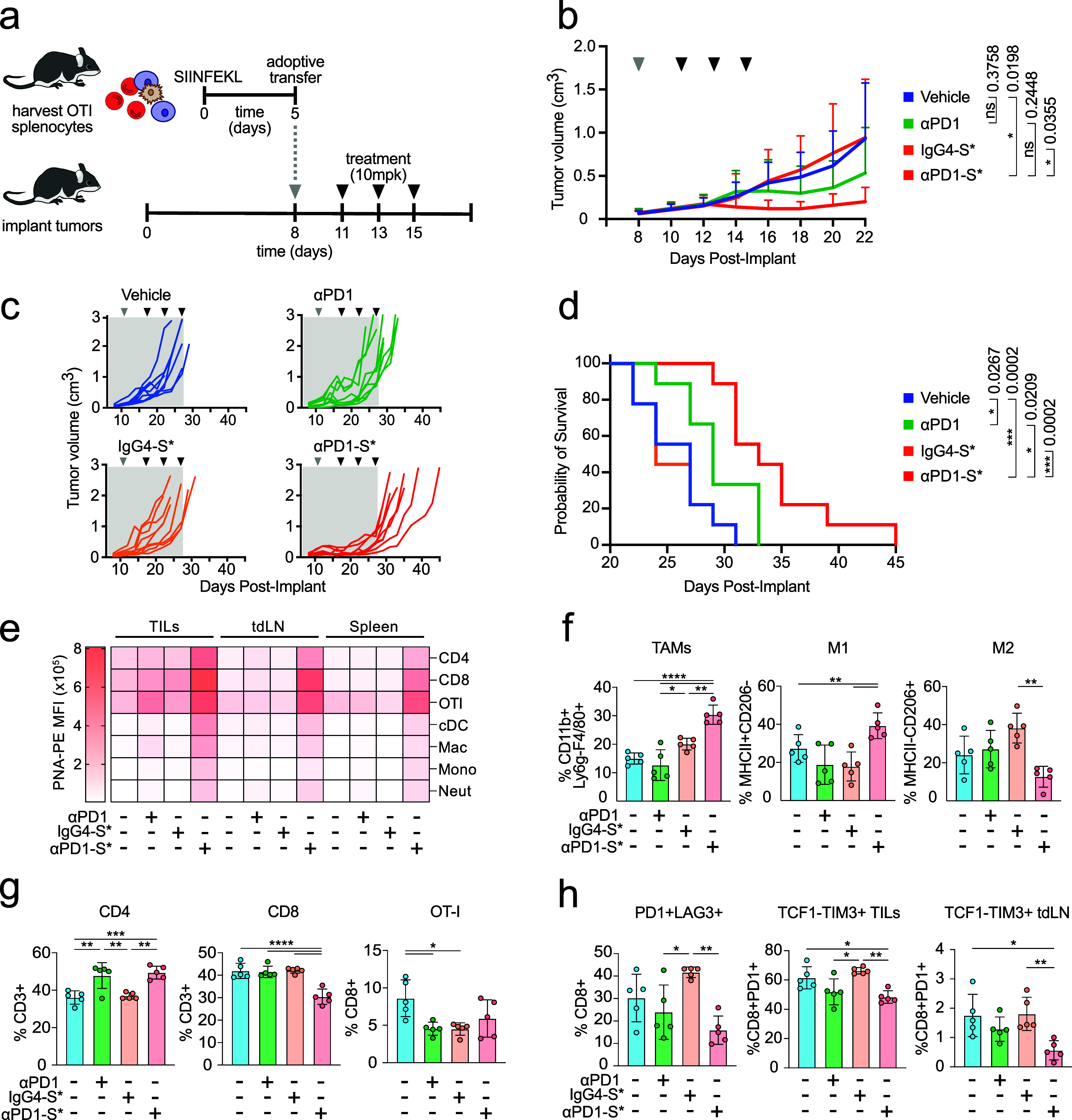
αPD1-S* enhances tumor inflammation and restricts growth in a murine melanoma model. (a) OT-I splenocytes were stimulated for 5 days *in vitro* using 200 nM SIINFEKL and adoptively transferred into C57BL/6 mice bearing 8-day-old subcutaneous B16OVA tumors on their flank. Mice were subsequently treated by intraperitoneal injection of vehicle or equimolar αPD1, αPD1-S*, or antibody isotype-sialidase (IgG4-S*) on the indicated days for three total treatments. (b) Tumor volume measured over time, plotted by individual in (c), with corresponding survival shown along a Kaplan–Meier plot in (d). (e) Quantitation of PNA staining of leukocytes from TILs, tdLN or spleen. (f) Proportions of tumor-associated macrophages (TAMs) expressed as percent CD11b+Ly6g-F4/80+ TILs. Within the TAM population, inflammatory (M1) subsets are expressed as percent MHCII+CD206-, while reparative (M2) subsets are expressed as percent MHCII-CD206+. (g) Frequencies of CD4+ and CD8+ TILs expressed as percent CD3+, OT-I frequencies expressed as percent CD8+. (h) Expression of checkpoint receptors PD1 and LAG3 as a percent of CD8+ TILs. Terminally exhausted T cell populations (TCF1-TIM3+) are expressed as percent PD1+CD8+ cells from TILs and tumor-draining lymph node (tdLN). Error represented as standard deviation *p < 0.05, **p < 0.01, ***p < 0.001, ****p < 0.0001 by one-way ANOVA followed by Tukey’s multiple comparisons test.

### Impact of αPD1-S on Immune Cells in the Tumor Microenvironment

To gain insights into the impact of αPD1-S* on immune responses, we analyzed leukocytes from tumors, tdLN or spleen, for their sialic acid content, relative abundance and effector phenotype using flow cytometry (Figure 5, Supplementary Figures S11 and S12). Notably, leukocytes from TILS, tdLN or spleen of mice treated with nontargeted IgG4-S* showed no increase in PNA staining compared to those from mice treated with αPD1, demonstrating a lack of off-target sialidase activity (Figure 5e). In contrast, CD4+, CD8+, and OT-I T cells from mice treated with αPD1-S* exhibited strong increases in PNA staining, consistent with targeting to PD1+ cells. Subtle increases in PNA staining were observed also for other leukocyte subsets, possibly due to basal PD1 expression on these cells^55–62^ or off-target effects mediated by cell–cell contacts with αPD1-S* on the surface of PD1+ T cells during antigen presentation.

Targeting sialidase to the tumors has been shown to restrict tumor growth by polarizing myeloid cells toward inflammatory phenotypes.^10^ We evaluated αPD1-S* for its impact on myeloid cells by measuring cell proportions in TILs, tdLN and spleen (Figure 5f,g, Supplementary Figure S12). Treatment with αPD1-S* significantly increased the frequency of tumor-associated macrophages (TAMs) compared to controls and within the TAM population, αPD1-S* shifted macrophage phenotypes toward M1-like, as indicated by an increase in MHCII+CD206- M1 macrophages and corresponding reduction in MHCII-CD206+ M2 macrophages (Figure 5f). This difference was also pronounced in spleen but not tdLN (Supplementary Figure S12) and is consistent with reports of a HER2-targeted sialidase that remodels the TME by skewing macrophage polarization toward M1.^10^ Notably, treatment with αPD1 increased CD8+ T cell proportions, while treatment with αPD1-S* moderately increased CD4+ T cells proportions within each compartment (Figure 5g). No major differences were observed in the proportions of OT-I (percent of total CD8) cells within these compartments.^63^

We examined the impact of αPD1-S* treatment on CD8 T cell exhaustion by analyzing phenotypic markers associated with exhaustion and regeneration. Specifically, we identified terminally exhausted CD8 T cells (TEX) as PD1+LAG3+TIM3+TCF1–, and progenitor exhausted CD8 T cells (PEX) as PD1+LAG3+TIM3–TCF1+, the latter representing a stem-like exhausted subset characterized by proliferative and self-renewal capacities.^50^ Although overall CD8 T cell frequencies were lower following αPD1-S* therapy, persistent CD8 T cells showed reduced proportions of PD1+LAG3+ cells relative to controls, indicating αPD1-S* limits progression toward terminal exhaustion. Indeed, αPD1-S* treatment significantly decreased TEX proportions within tumors and tumor-draining lymph nodes (tdLN) (Figure 5h, Supplementary Figure S13). Conversely, PEX populations were enriched, particularly within TILs and tdLN, suggesting enhanced proliferative and regenerative potential following αPD1-S* treatment (Figure 5h, Supplementary Figure S13). Collectively, these findings reinforce prior evidence that therapeutic targeting of the PD1 axis can restore progenitor T cell functionality and support durable antitumor immunity.^64,65^ .^66^

Thus, αPD1-S* likely enhances tumor control by limiting terminal differentiation of CD8 T cells and preserving a progenitor-like, functionally competent T cell population.

## Discussion

Sialidase therapies targeting hypersialylation in the TME have emerged as a promising strategy for cancer immunotherapy. Sialidases therapies targeting tumors,^10,27,28,30,31^ T cells,^31,67^ and NK cells^30^ have collectively demonstrated improved tumor control across diverse models. Sialoglycans are well documented as ligands for inhibitory Siglecs that can suppress immune responses by being recruited to the immunological synapse between immune cells and target cells. Sialoglycans on T cells and APCs limit immune synapse formation and inhibit signaling through the costimulatory receptor CD28, which is essential for effective PD1 blockade.^32–37^ For these reasons, we developed αPD1-S, a PD1-blocking antibody conjugated to sialidase to enhance immune responses by removing inhibitory sialoglycans and potentiating PD1 checkpoint blockade. Here we show that by selectively removing inhibitory sialoglycans on PD1+ T cells, αPD1-S enhances T cell effector function and improves tumor control.

αPD1-S was rapidly assembled as a modular conjugate and demonstrated ≥1000-fold selectivity for PD1+ T cells with efficient targeting *in vitro* and *in vivo* (Figures 2 and 3). Functionally, αPD1-S exerted distinct effects across T cell effector types. While it did not enhance cytotoxicity in OT-I cells activated *in vitro* (Supplementary Figure S6), it significantly improved tumor cell killing by chronic, LCMV exhausted P14 T cells (Figure 4), suggesting desialylation preferentially benefits hypofunctional T cells. These findings are consistent with reports that Siglec-7/-9 can be trogocytosed onto T cells from myeloid cells,^68^ raising the possibility that P14 cells similarly acquire the functional paralog Siglec-E *in vivo*. Taken together, our results support a role for αPD1-S in degrading *cis*-sialoglycan ligands of inhibitory Siglecs that constrain T cell activation.^15,17,22,69,70^

In preliminary experiments, we evaluated the ability of systemic αPD1 and αPD1-S to reduce the growth of tumors produced by MC38, CT26, and B16F10 cells, and saw minimal suppression by either agent. To evaluate the enhanced activity of αPD1-S compared to αPD1, we utilized the B16OVA model previously demonstrated by Bull et al.^24^ to be sensitive to inhibitors of sialoglycan biosynthesis, coupled with adoptive transfer of PD1-expressing, antigen-specific OT-I cells. Moreover, we elected to use the αPD1-S* conjugate containing the R309A variant with reduced sialidase activity, since we had seen strong off-target desialylation of cells using the wild type αPD1-S (Figure 3f,g), similar to off-target desialylation seen with a tumor-targeted antibody sialidase conjugate reported by Gray et al.^28^

*In vivo*, αPD1-S* significantly reduced B16OVA tumor burden and extended survival, and improved survival over αPD1 therapy alone, showing the synergistic impact of the targeted sialidase (Figure 5a-d). Increases in PNA staining were detected on myeloid cells within tumor-infiltrating lymphocytes, as well as in tdLN and spleen, suggesting low basal PD1 expression on myeloid subsets^55–62^ or off-target activity of αPD1-S* resulting from contact with PD1+ T cells during antigen presentation (Figure 5e). Beyond its direct effects on T cells, αPD1-S* reprogrammed the tumor microenvironment by shifting tumor-associated macrophages (TAMs) from an immunosuppressive M2 phenotype to an inflammatory M1 phenotype. This myeloid remodeling mirrors findings with HER2-targeted sialidases, suggesting a conserved mechanism in which sialoglycan degradation facilitates myeloid activation and promotes antitumor immunity^10^ (Figure 5f).

The emerging role of sialoglycans as critical regulators of immune suppression suggests glycoimmune checkpoints represent an underexplored yet actionable therapeutic target. The potential of targeting the sialic acid axis in cancer is underscored by a report showing that direct injection of a sialyltransferase inhibitor into B16OVA tumors, likely reducing both tumor and immune cell sialic acids, induced complete regression of tumors mediated by endogenous CD8 T cells.^24^ Moreover, cured animals further rejected tumor cells upon subsequent rechallenge, demonstrating they had acquired tumor-specific, adaptive immunity.^24^ Thus, novel strategies for targeting the sialic acid axis hold great promise as an adjunct to future cancer therapy.^27–31^ In addition to sialidase based approaches, recent sialic acid “blocking” molecules termed antibody-lectin chimeras (AbLecs)^71^ have demonstrated enhanced efficacy over tumor-targeted sialidases, reinforcing the concept of “professional Siglec ligands”—a subset of sialylated glycoconjugates with high affinities for inhibitory Siglecs.^7,72–75^ While αPD1-S promotes broad desialylation to enhance T cell function, a more refined approach targeting specific Siglec ligands, such as Siglec-7/-9 ligands on T cells, may yield additional therapeutic advantages. Importantly, unlike tumor-directed sialidases, which rely on heterogeneous antigen expression, αPD1-S provides a generalized strategy for refractory tumors infiltrated by PD1+ lymphocytes. Together, these findings establish αPD1-S as a rationally designed glycoimmune checkpoint therapy that expands the concept of targeted sialidase modalities and amplifies the efficacy of PD1 blockade by overcoming sialoglycan-mediated immune suppression.

## Supplementary Material







## Notes

Detailed experimental methods (PDF)
Additional data, including SDS-PAGE analyses, contour plots, structures, sialic acid analyses, flow cytometry analyses, growth curves, tumor volume data, ELISA analyses, gating strategies, and frequencies of immune cell subsets; antibodies used in this study; and amino acid sequences used in this study (PDF)
Transparent Peer Review report available (PDF)
