# Supplementary material for: Targeting Sialidase to PD1 Enhances T cell Function and Tumor Control
Source: ACS Cent Sci. 2025 Jul 4;11(8):1417–27. doi: 10.1021/acscentsci.5c00510 (PMC12395300; doi:10.1021/acscentsci.5c00510)
Supplement: Supplementary file 1 [file oc5c00510_si_001.pdf]

Targeting Sialidase to PD-1 Enhances T cell Function and Tumor Control

Brett M Garabedian[1], Eleanor E Bashian[1,2], Xiaoshuang Wang[1],
Andrew J Thompson[1], James C Paulson[1*]

1. Department of Immunology and Microbiology, The Scripps Research Institute, La Jolla, California 92037, United States

2. Department of Molecular and Cellular Biology, The Scripps Research Institute, La Jolla, California 92037, United States

*Corresponding author
Email: jpaulson@scripps.edu
Project funding: NIAID AI171628

Materials and Methods

Animal Procedures and General Housing. All mouse experiments were approved by the Institutional Animal Care and Use Committee (IACUC) at The Scripps Research Institute, following NIH guidelines. Mice were maintained under specific pathogen–free conditions in individually ventilated cages. Temperature in the animal facility was kept at 20–23°C with 40–60% relative humidity and a 12 h light–dark cycle. Bedding was changed twice per week, and environmental enrichment was provided. Standard rodent chow and water were supplied ad libitum. Both male and female mice between six and twelve weeks of age were used unless otherwise stated, and group sizes varied as indicated in figure legends. Health checks were performed weekly. Randomization of mice was conducted prior to treatment in all cases.

Mice. C57BL/6 (wild-type) mice were bred in-house. OT-I mice (strain B6.*129S6-Rag2tm1Fwa*Tg(TcraTcrb)1100Mjb, CD45.2+/+) were purchased from Taconic Biosciences. OT-I+/− CD45.1+/− mice were bred in-house using C57BL/6J (CD45.1+/+) and OT-I mice (CD45.2+/+). For chronic T-cell exhaustion studies, P14 TCR-transgenic mice [1] (generously provided by the laboratory of John Teijaro at Scripps Research) were used as donors for adoptive transfer experiments. Balb/c were purchased from Jackson labs and similarly housed.

Statistical Analysis. Data are generally presented as mean ± standard deviation (s.d.). GraphPad Prism software was used for all calculations. One-way or two-way analyses of variance (ANOVA) with Tukey's corrections were applied to compare multiple groups, while unpaired two-tailed t-tests were used for pairwise comparisons when applicable. Survival curves were analyzed by the log-rank (Mantel–Cox) test. Significance levels are designated in figure legends as $P < 0.05$ (*), $P < 0.01$ (**), $P < 0.001$ (***), or $P < 0.0001$ (****). The exact numbers of replicates, animals per group, and statistical tests are detailed for each figure or table.

Production of Sortase A (SrtA)*.* The SrtA variant 8M[2] was subcloned into a pET23 plasmid upstream of a C-terminal TEV protease cleavage site and a maltose-binding protein (SrtA-TEV-MBP-6xHIS). BL21(DE3) cells were transformed with the SrtA-TEV-MBP-6xHIS construct and selected on LB agar containing 100 µg/mL carbenicillin. A single colony was grown overnight in 5 mL LB medium with carbenicillin. This starter culture was used to inoculate 1–2 L of TB–carbenicillin, which was grown at 37°C to an OD600 of 0.6–0.8. Protein expression was induced by adding 1 mM IPTG, and the temperature was reduced to 16–20°C for 16 h. Bacterial pellets were harvested by centrifugation (4,000 × g, 15 min, 4 °C), resuspended in lysis buffer (50 mM Tris, 300 mM NaCl, 10 mM imidazole, pH 7.5), and disrupted by sonication on ice. Lysates were clarified by centrifugation at 10,000 × g for 30 min at 4 °C. Clarified lysates were applied to Ni-NTA resin (Qiagen) equilibrated with lysis buffer. After washing with 20 column volumes of wash buffer (50 mM Tris, 300 mM NaCl, 20 mM imidazole, pH 7.5), SrtA-TEV-MBP-6xHIS was eluted with elution buffer (50 mM Tris, 300 mM NaCl, 250 mM imidazole, pH 7.5). Elution fractions were concentrated using 10 kDa MWCO centrifugal filters (Millipore), then buffer exchanged into TEV cleavage buffer (50 mM Tris, 300 mM NaCl, 1 mM DTT, pH 7.5). TEV protease (10 mol%) was added and incubated overnight at 4°C with gentle stirring. Cleavage efficiency was monitored by SDS–PAGE. The cleaved protein was purified by size-exclusion chromatography on a Superdex 200 column (Cytiva) equilibrated with 50 mM Tris, 300 mM NaCl (pH 7.5). Fractions containing pure SrtA were pooled, concentrated, and stored at −80°C.

Production of Salmonella typhimurium NanH Sialidases (Wild-Type and R309A). Genes encoding wild-type NanH[3] and the R309A mutant were synthesized (Twist Biosciences) and subcloned into pET-23 vectors, providing a C-terminal 6×His tag. BL21(DE3) cells harboring pET-23–NanH (WT or R309A) were grown in TB-Carbenicillin (50 µg/mL) to OD600 ≈ 0.6, induced with 1 mM IPTG, and cultured at 18°C for 16 h. Cells were pelleted, resuspended in 20 mM Tris (pH 7.5), 150 mM NaCl, and lysed by sonication. After clarification (10,000 × g, 30 min, 4 °C), the supernatant was bound to Ni–NTA resin. The column was washed with 30 mM imidazole, and sialidase was eluted with 300 mM imidazole. Eluted fractions were buffer-exchanged into 20 mM Tris (pH 7.5), 150 mM NaCl, and further purified by Superdex 200 SEC. Enzyme concentrations were determined by A280.

Production of anti-PD1 (αPD1). Variable regions encoding murine anti-PD1 (armenian hamster clone J43), human anti-PD1 (clone 409A11 derived from pembrolizumab) or IgG4 isotype (derived from Motavizumab specific for respiratory syncytial virus (RSV)) were engineered onto a human IgG4 Fc containing the LPETG sortase recognition motif at the heavy- or light-chain C terminus. Coding sequences for light and heavy chains were fused by two consecutive self-cleaving peptides [4] P2A-T2A, and expressed as a polycistronic construct to ensure 1:1 heavy light ratios. Antibodies were expressed in ExpiCHO cells by transient transfection and cultured for 10 days. Harvested supernatants were pH-adjusted to pH 8.0 using 1M Tris HCl and loaded onto a Protein A column (Cytiva), washed with PBS, and eluted with 100 mM citrate (pH 3.0), which was immediately neutralized by the addition of 10%v/v 1M Tris HCl pH 8.0. Prior to conjugation by SrtA, antibody fractions were concentrated to ~70µM and snap frozen.

Sortase Conjugation Reaction. The conjugation reaction was performed in 50 mM Tris, 150 mM NaCl, 100 mM $CaCl_2$ (pH 8.2). Typically, 10-20 mg antibody (30 µM final) was mixed with 20 molar equivalents of poly-glycine–tagged sialidase (600 µM final) and 5 molar equivalents of SrtA (150 µM final), in that order. The reaction (volume 1–3 mL) was incubated in 50ml conical tube placed into a water bath at 37°C for 1 minute, swirling at 100RPM. After 1 minute, the reaction was diluted 10-fold by the addition of reaction quenching buffer (50 mM Tris, 150 mM NaCl, 20mM EDTA). Upon completion, the reaction mixture was purified by protein A (capturing αPD1 and αPD1-sialidase), followed by Ni-NTA IMAC (capturing αPD1-S). Purified αPD1–sialidase (αPD1-S) was buffer exchanged into DPBS, concentrated to 2mg/ml, snap frozen and stored at −80°C until further use.

PD1 ELISA. ELISA plates (Maxisorb Nunc) were coated with 100µl (5µg/ml in DPBS) recombinant hPD1 (R&D systems 8986-PD) or mPD1 (R&D systems 9047-PD) overnight at 4˚C. Coated plates were blocked for 1h at ambient temperature with DPBS containing 2% BSA. Blocked plates were incubated with serial dilutions of αPD1 or αPD1-S in DPBS for 1h at ambient temperature. Plates were then washed three times with DPBS containing 0.1% Tween-20 (DPBST). To washed plates was added 100µl of anti-human IgG-HRP diluted 1:5000 (Promega W4031) for 1h at ambient temperature. Plates were washed three times with DPBST, followed by the addition of 50µl HRP substrate (Thermo 34028) for 10 minutes. Peroxidase activity was quenched by the addition 50µl 2N H_2SO_4. Binding to PD1 was determined by measuring absorbance at 450nM.

Sialidase Activity. To measure sialidase activity, 4-methylumbelliferyl–N-acetylneuraminic acid (4-MUNANA; Sigma) was dissolved in DMSO at 100 mM and stored at −20°C. Reactions (100 µl) in a 96-well black plate contained 0.05–1 mM 4-MUNANA in 50 mM sodium acetate buffer (pH 6.5) and dilutions of sialidase (e.g., 5–50 nM). Plates were incubated at 37°C for 30 min, and fluorescence (excitation 365 nm, emission 450 nm) was measured every 5 min on a plate reader (Tecan). Activity was quantified against a standard curve of 4-methylumbelliferone (0–10 µM) prepared in the same buffer.

Jurkat Cell Desialylation. Jurkat cells (WT and PD1+GFP+) were maintained in RPMI + 10% FBS and 1% pen–strep. To investigate selective desialylation, WT and PD1+GFP+ cells were suspended in DPBS containing calcium, magnesium (Thermo 14040133) and 0.1% BSA and mixed in equal numbers ($1x10^5$ each) in a U-bottom 96-well plate. αPD1–sialidase was added at the indicated concentration for 1 h at 37°C without shaking, after which cells were washed twice with cold PBS and stained with biotinylated lectins precomplexed with streptavidin-PE (Biolegend) (SNA, MAA, PNA; Vector Labs, 2 µg/mL) for 30 min on ice. After a final wash, samples were analyzed by flow cytometry to compare sialic acid levels on WT and PD1+ subsets.

Immunofluorescence Microscopy. Mice harboring CT26 tumors measuring $500mm^3$ were treated by a single intraperitoneal injection (10mg/kg) of αPD1, equimolar sialidase or αPD1-sialidase. Mice were euthanized 24h post-injection and tissues resected, fixed, and frozen in OCT mounting medium**.** Cryosections (5–10 µm) were mounted on Superfrost Plus slides (Fisherbrand) and fixed for 10 min in 4% paraformaldehyde (Electron Microscopy Sciences). After permeabilization with 0.1% Triton X-100, slides were blocked with 1% normal goat serum (Jackson ImmunoResearch) in PBS for 30 min. Sections were then stained for 1h at ambient temperature with anti-CD3 or fluorescent lectins (PNA, MAA). DAPI (Thermo Fisher) was used at 300 nM to label nuclei. Slides were mounted with ProLong Diamond (Invitrogen) and cured overnight. Images were captured on a Zeiss Axio Imager fluorescence microscope using consistent exposure settings. Post-acquisition processing (brightness/contrast) was performed in ImageJ, applying identical parameters across all samples within an experiment. Tumor draining lymph nodes from these same mice were dissociated into single cells, stained with PNA-PE and lineage markers for CD8 (CD3, CD8) and CD4 (CD3, CD4) T cells, and B cells (CD19), and analyzed by flow cytometry using t-distributed stochastic neighbor embedding (t-SNE).

Harvesting Splenocytes. Spleens were dissected under sterile conditions and placed in cold RPMI 1640 supplemented with 10% FBS and 1% penicillin–streptomycin. They were transferred to a 40 µm cell strainer and gently dissociated using the plunger of a 3 mL syringe. Red blood cells were lysed in ACK buffer (Thermo Fisher) for 1–2 min at ambient temperature, quenched with RPMI + 10% FBS, and washed twice by centrifugation (300 × g, 5 min). Cells were counted and resuspended in complete RPMI-1640 medium supplemented with 10% fetal bovine serum (FBS), 1% penicillin-streptomycin (Pen-Strep), 50 µM β-mercaptoethanol, 10 mM HEPES, 1 mM sodium pyruvate, 1X non-essential amino acids (NEAA), and 2 mM GlutaMAX.

OT-I Activation. OT-I splenocytes were seeded in 6-well plates at a density of 1×10^6 cells per mL and stimulated with the indicated concentration of SIINFEKL peptide at 37°C in 5% CO_2 for five days. The culture medium was supplemented with 50 U/mL IL-2 to support T cell activation

and expansion. Partial medium changes were performed on days 2–3 to maintain optimal cell growth and cytokine availability.

Mouse OT-I T-Cell Re-Stimulation. After the 5-day SIINFEKL activation, OT-I T cells were harvested, counted, and replated at $1x10^3$ per well in a 96-well plate. They were restimulated with plate-bound anti-CD3 (5µg/ml) ± αPD1, ± free sialidase, or ± αPD1–S for 24 h (0.1nM or 1nM treatment). Brefeldin A (BioLegend) was added during the last 4–6 h to allow intracellular cytokine accumulation. Cells were then stained with lectins (MAA, PNA) and antibodies for IFNγ, TNFα, or granzyme B (after fixation/permeabilization), and analyzed by flow cytometry.

Infection with LCMV clone 13. To induce chronic infection, C57BL/6 mice were infected intravenously via the lateral tail vein with $2x10^6$ plaque-forming units (PFU) of LCMV Clone 13, prepared as an inoculum in 200 µl sterile phosphate-buffered saline (PBS). In certain experiments, $1x10^5$ P14 T cells were adoptively transferred one day before infection. Mice were monitored daily for signs of clinical disease or excessive weight loss (>20% initial body weight). Analyses were performed on days 15–25 post-infection, as indicated in the figure legends.

Activation of LCMV exhausted T cells. Splenocytes from d15 LCMV infected mice were prepared and restimulated in vitro with GP33 or other LCMV peptides (NP205, GP61, GP276, NP396; 1µg/ml final for each) for 72 h in the presence or absence of αPD1, free sialidase, or αPD1–S. Following treatment, conditioned supernatants were diluted 100-fold in DPBS and assayed for INFy production (Biolegend 430804) according to the manufacturer's protocol.

LCMV P14 T-Cell Killing. Chronic exhaustion was induced by transferring P14 T cells ($2x10^5$) into C57BL/6 mice 24h before LCMV Clone 13 infection. On day 25 post-infection, splenocytes were harvested and T cells isolated by negative e selection (STEMcell Technologies, 19851). PD1+P14+ cells were sorted using Bigfoot spectral cell sorter (Thermofisher). These exhausted T cells were then co-cultured at a 1:1 ratio with B16 cells stably expressing GP33 (B16GP33, generously provided by the laboratory of John Teijaro) in complete RPMI for 72 h, ± αPD1, ± sialidase, or ± αPD1–S at 100pM final concentration. Cytotoxicity was quantified by an LDH release kit (Pierce), measuring absorbance at 490 nm (LDH substrate) and correcting for baseline release from untreated cells.

OT-I:B16OVA Co-Cultures. After 5-day activation (200nM SIINFEKL initial) , OT-I cells were cocultured with B16OVA-GFP cells (generously provided by the laboratory of Peng Wu at Scripps Research), seeded at a 1:1 ratio in 96-well plates ($5x10^4$ each). Cell viability was determined in real time over 72h using an Incucyte S3 Live Cell Analysis Instrument, normalizing for 100% viability against wells containing B16OVA-GFP cells only.

Tumor Implantation. Tumor cells were cultured in DMEM (B16OVA) or RPMI (CT26) + 10% FBS, then lifted with 0.05% trypsin–EDTA, washed twice in PBS, and counted. $1x10^6$ cells in 50µl serum-free RPMI were injected subcutaneously (27G needle) into the right flank of male C57BL/6 mice (B16OVA tumors) or female Balb/c (CT26 tumors) mice. Tumors were measured by calipers every 2–3 days; volume was calculated as (length × width^2)/2. Mice were euthanized when tumors approached 2,000 mm^3, or upon ulceration.

Adoptive T-Cell Transfer. 5-day 200nM SIINFEKL-activated OT-I cells ($1x10^6$) were administered intravenously (200 µl RPMI) on day 8 post-tumor inoculation. Treatments started on day 11 with PBS (vehicle), αPD1 (10 mg/kg), IgG4–sialidase (isotype control), or αPD1–sialidase (αPD1–S). Dosing was repeated on days 13 and 15 at the same concentration (i.p. injection, 10 mg/kg). Tumor size was documented, and survival was monitored until humane endpoints were reached. At the study endpoint, tumors were excised and processed into single cell suspensions for flow cytometry

Tissue Processing for Flow Cytometry. Tumors were dissected under sterile conditions and placed in cold RPMI 1640 supplemented with 10% FBS and 1% penicillin–streptomycin. They were transferred to a 40 µm cell strainer and gently dissociated using the plunger of a 3 mL syringe. Red blood cells were lysed in ACK buffer (Thermo Fisher) for 1–2 min at ambient temperature, quenched with RPMI + 10% FBS, and washed twice by centrifugation (300 × g, 5 min). Pellets were resuspended in PBS + 2% FBS + 2mM EDTA (FACS buffer).

Antibody Staining for Flow Cytometry. Samples were incubated with a viability dye (Ghost Violet 510, Tonbo Biosciences) in PBS for 10 min at ambient temperature, then washed once with DBPS+0.1%BSA. Fc receptors were blocked with anti-CD16/CD32 (BioLegend) for 10 min. Surface markers were stained for 30 min on ice using fluorophore-conjugated antibodies (see Supplementary Table, typically 0.25–2 µg/test). Intracellular staining (e.g., IFNγ, granzyme B, TCF1) was performed after fixation/permeabilization with Foxp3 / Transcription Factor Staining Kit, eBioscience. Compensation was set using single-color beads or cells.

Data Acquisition and Analysis. Stained samples were acquired on a Bio-Rad ZE5 or Cytek Aurora spectral flow cytometer, typically collecting ≥20,000 events per sample. PMT voltages or spectral unmixing settings were optimized with appropriate controls. Data were analyzed in FlowJo (BD Biosciences), using hierarchical gating (e.g., forward and side scatter, viability dye, CD45, T-cell or myeloid markers, PD1, etc.). Representative gating strategies are provided in Supplementary Figures S6–S8, S11–S12. Graphs of cell frequencies, mean fluorescence intensities (MFIs), and cytokine production were generated in GraphPad Prism.

Pharmacokinetic and Anti-Drug Antibody (ADA) Analysis. Mice received a single intraperitoneal injection (10 mg/kg) of αPD1, αPD1–sialidase, or an isotype–sialidase control. Blood samples (50–200 µl) were collected via retroorbital bleeding using heparanized capillaries (e.g., 1 h, 3 h, 24 h, 48 h, 72 h, 120 h, 168 h, 336 h). Serum was separated by centrifugation (2,000 × g, 10 min, 4°C), diluted 10-fold into DPBS and stored at −80 °C. Sialidase activity was measured by 4-MUNANA assay using 10 µl of diluted serum in a 100 µl reaction volume. One-phase exponential decay curves were fit in GraphPad Prism to estimate half-lives. Anti-drug antibodies (ADAs) were detected by coating 96-well ELISA plates with 5 µg/mL of sialidase, αPD1 or IgG4 (motavizumab[3]). After blocking with 1% BSA in PBS, serum samples were serially diluted and added. Plates were washed three times in DPBST and bound antibodies were detected with an HRP-conjugated secondary (anti-mouse IgG-HRP, Thermo 31430). Plates were developed with TMB substrate (Thermo 34028), stopped with 1N H_2SO_4, and absorbance read at 450 nm. ADA titers were the highest dilution yielding ≥2× blank OD values.

References

(1) Pircher, H.; Bürki, K.; Lang, R.; Hengartner, H.; Zinkernagel, R. M. Tolerance induction in double specific T-cell receptor transgenic mice varies with antigen. *Nature* **1989**, *342* (6249), 559-561. DOI: 10.1038/342559a0.
(2) Chen, L.; Cohen, J.; Song, X.; Zhao, A.; Ye, Z.; Feulner, C. J.; Doonan, P.; Somers, W.; Lin, L.; Chen, P. R. Improved variants of SrtA for site-specific conjugation on antibodies and proteins with high efficiency. *Sci Rep* **2016**, *6*, 31899. DOI: 10.1038/srep31899 PubMed.
(3) Gray, M. A.; Stanczak, M. A.; Mantuano, N. R.; Xiao, H.; Pijnenborg, J. F. A.; Malaker, S. A.; Miller, C. L.; Weidenbacher, P. A.; Tanzo, J. T.; Ahn, G.; et al. Targeted glycan degradation potentiates the anticancer immune response in vivo. *Nat Chem Biol* **2020**, *16* (12), 1376-1384. DOI: 10.1038/s41589-020-0622-x.
(4) Liu, Z.; Chen, O.; Wall, J. B. J.; Zheng, M.; Zhou, Y.; Wang, L.; Ruth Vaseghi, H.; Qian, L.; Liu, J. Systematic comparison of 2A peptides for cloning multi-genes in a polycistronic vector. *Scientific Reports* **2017**, *7* (1), 2193. DOI: 10.1038/s41598-017-02460-2.
