# Supplementary material for: Targeting Sialidase to PD1 Enhances T cell Function and Tumor Control
Source: ACS Cent Sci. 2025 Jul 4;11(8):1417–27. doi: 10.1021/acscentsci.5c00510 (PMC12395300; doi:10.1021/acscentsci.5c00510)
Supplement: Supplementary file 2 [file oc5c00510_si_002.pdf]

Targeting Sialidase to PD-1 Enhances T cell Function and Tumor Control

Brett M Garabedian[1], Eleanor E Bashian[1,2], Xiaoshuang Wang[1], Andrew J Thompson[1], James C Paulson[1*]

1. Department of Immunology and Microbiology, The Scripps Research Institute, La Jolla, California 92037, United States

2. Department of Molecular and Cellular Biology, The Scripps Research Institute, La Jolla, California 92037, United States

Table of Contents

Supplemental Figure S1. Reducing SDS-PAGE analysis of recombinant proteins and optimization of anti-PD1-sialidase (αPD1-S) conjugation.

Supplemental Figure S2. Structural and functional characterization of wild-type and mutant (R309A) ST sialidase enzyme variants used in αPD1-S conjugates.

Supplemental Figure S3. Reduction of sialoglycans on PD1-expressing Jurkat T cells after treatment with αPD1-S.

Supplemental Figure S4. PD1-dependent sialic acid removal from differentially activated OT-I splenocytes, detected by PNA lectin staining.

Supplemental Figure S5. Analysis of T cell exhaustion markers (PD1, LAG3, Ly108, TIM3) on OT-I cells stimulated with varying concentrations of SIINFEKL peptide.

Supplemental Figure S6. Gating strategy for flow cytometry analyses described in Figure 4d-f (main text).

Supplemental Figure S7. Co-culture of activated OT-I cells with B16OVA-GFP tumor cells treated with anti-PD1, αPD1-S, or IgG4-S, demonstrating tumor cell viability and sialic acid removal.

Supplemental Figure S8. Gating strategy for cell-killing experiments described in Figure 4i-j (main text).

Supplemental Figure S9. Adoptive transfer of OT-I splenocytes into tumor-bearing mice followed by treatment with anti-PD1, αPD1-S, or IgG4-S and analysis of tumor growth and survival.

Supplemental Figure S10. Pharmacokinetic and immunogenicity analysis of αPD1-S, anti-PD1, and IgG4-S conjugates in mice, assessing enzyme activity and anti-drug antibody (ADA) responses.

Supplemental Figure S11. Representative gating strategies for immune cell subset analyses (T cells and myeloid cells) in tumor, spleen, and tumor-draining lymph node.

Supplemental Figure S12. Frequencies of immune cell subsets identified in tumor-infiltrating lymphocytes (TILs), tumor-draining lymph nodes (tdLN), and spleen.

Supplemental Figure S13. Analysis of PD1+LAG3+ cells and TCF1/TIM3-expressing subsets within CD4+ and CD8+ PD1+ T cell populations in TILs and tdLN.

Supplemental Table S1. Fluorescently labeled antibodies used in this study.

Supplemental Table S2. Amino acid sequences for proteins used in this study.

Supplemental Figures (S1-S13)

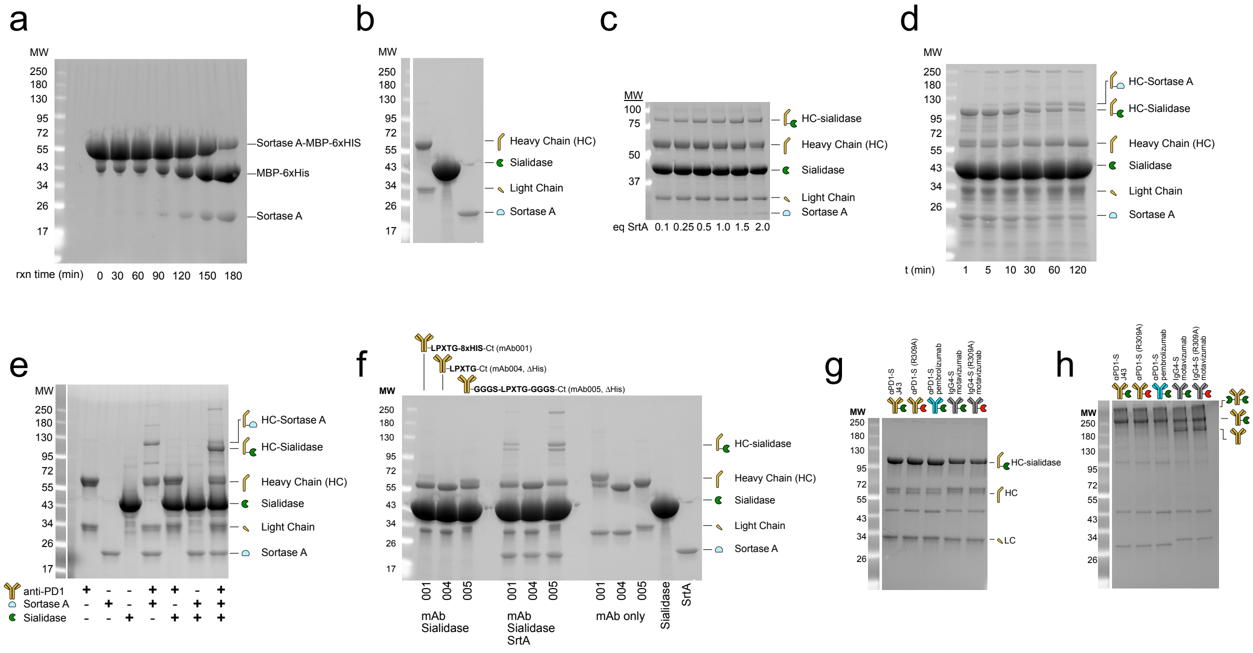

Figure S1. (**a-h**) Reducing SDS-PAGE analysis of recombinant proteins produced in this study. (**a**) MBP-tagged Sortase A (SrtA) is digested with 0.1 molar equivalents TEV protease and digested for 2h at 37˚C; reaction products are shown at 30min intervals. Liberated SrtA is further isolated by size-exclusion chromatography (not shown). (**b**) Purified anti-PD1, ST sialidase[1] and SrtA used for αPD1-S construction. (**c**) αPD1-S reaction optimization, indicating band intensity corresponding to HC-sialidase that increases with increasing concentrations of SrtA. (**d**) αPD1-S reaction optimization, indicating band intensity corresponding to HC-sialidase that is reduced over time, with concomitant emergence of a side products over time. (**e**) αPD1-S reaction optimization and side product characterization. Purified proteins (anti-PD1, 30μM; ST Sialidase, 300μM; SrtA, 150μM) are reacted in uniquely paired combinations, indicting the emergence of a ~100kDa impurity resulting from off-target SrtA reactivity with C-terminally LPETG-tagged anti-PD1 in the absence of N-terminally polyglycine-tagged ST sialidase (lane 4). (**f**) Optimization of C-terminal LPETG linker region on anti-PD1. Purified proteins (anti-PD1, 30μM; ST Sialidase, 600μM; SrtA, 150μM) are reacted in uniquely paired combinations, indicating the emergence of ~100kDa bands corresponding to HC-sialidase and HC-sortase, each intensified by the addition of GGGS linkers on either side of the SrtA recognition sequence. (**g,h**) Purified αPD1-S (J43, anti-mouse; Pembrolizumab, anti-human; and Motavizumab IgG4 isotype) equipped with WT sialidase or the R309A variant characterized in Supplementary Figure S5.

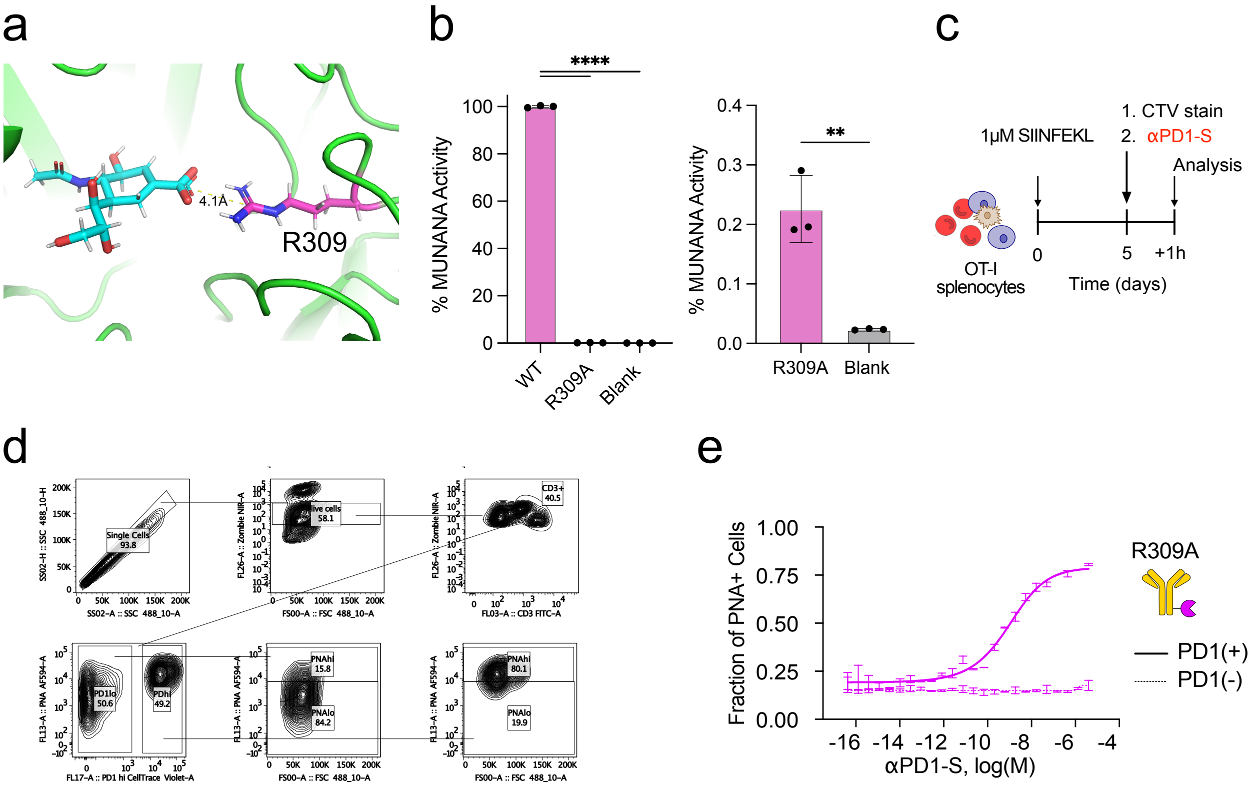

Figure S2. (**a**) Co-crystal structure (PDB 7AEY) of *S. typhumirium* NanH sialidase in complex with the sialic acid analog, isocarba-DANA, indicating residue R309 identified by alanine scanning (this study) to afford a mutant (R309A) with ~0.2% activity of wild-type (WT), as determined using the fluorogenic sialidase substrate 4-MUNANA in **b**. (**c**) Splenocytes from OT-I mice were stimulated with 1μM SIINFEKL for 5d and subsequently stained with CellTrace Violet and mixed 1:1 with naïve, freshly isolated (PD1-lo) splenocytes. Cell mixtures were separately treated with titrations of αPD1-S equipped with wild-type ST sialidase or the activity variant R309A, and subsequently analyzed for sialic acid content as determined by PNA staining and flow cytometry. (**d**) Gating strategy for sialic acid analysis of cell mixtures. (**e**) PNA lectin curves indicating the extent of sialic acid degradation by αPD1-S*, indicating retention of on-target sialidase activity by the R309A sialidase variant. For **b** (left) error bars represent standard deviation where p-values were determined by matched one-way ANOVA followed by Tukey's multiple comparisons test, where ****p ≤ 0.0001. For **b** (right) Unpaired student's t-test was used where **p≤0.01.

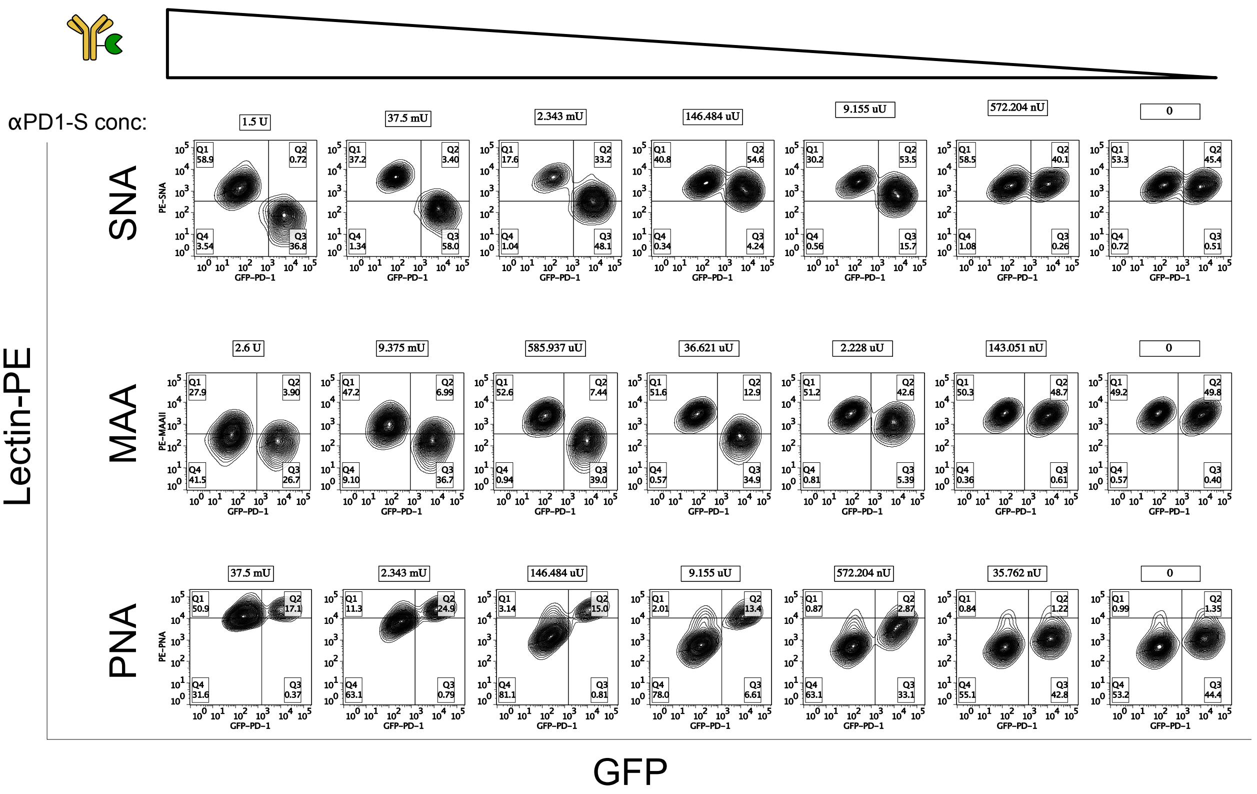

Figure S3. PD1(-)GFP(-) and PD1(+)GFP(+) Jurkat T cells were mixed in equal parts and treated with αPD1-S. Representative contour plots demonstrating reduced detection of sialoglycans by SNA, MAA and PNA on PD1(+)GFP(+) cells following treatment with αPD1-S at the indicated concentration.

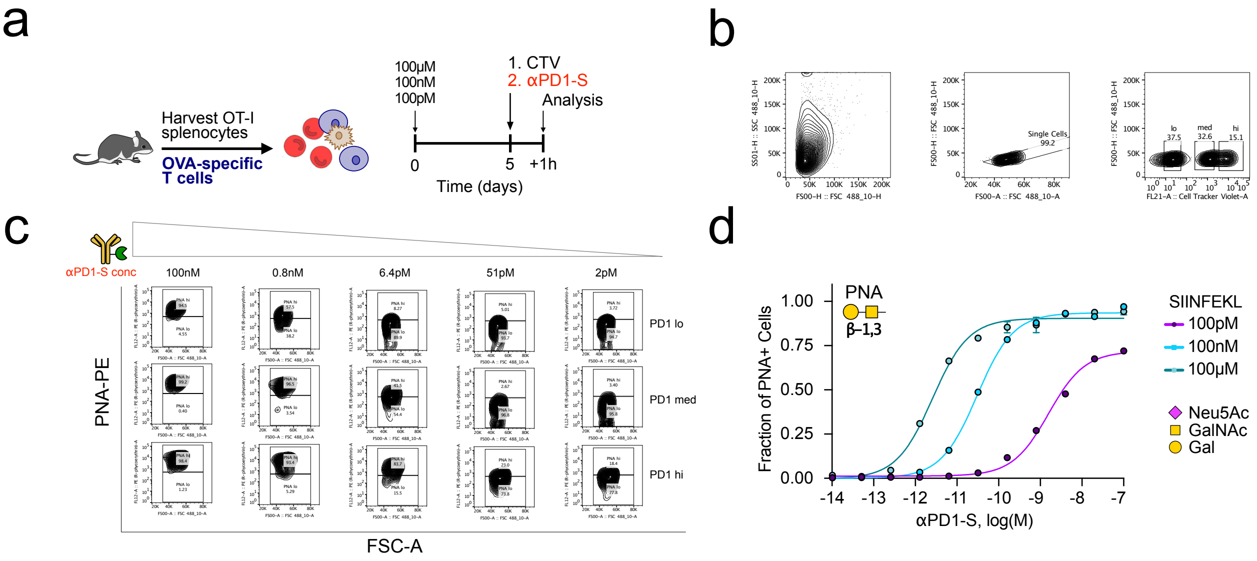

Figure S4. (**a**) OT-I splenocytes were generated by stimulation with the 100pM, 100nM or 100µM SIINFEKL peptide for 5d to afford PD1-lo, PD1-med, and PD1-hi, which were subsequently stained with 0µM, 0.1µM or 1µM CellTrace Violet, respectively. (**b,c**) OT-I cells (CTV_{lo}, CTV_{med}, CTV_{hi}) were then mixed at a ratio of 1:1:1 before treatment with αPD1-S for 1h at 37˚C and subsequent sialic acid analysis as determined by PNA staining and flow cytometry. (**d**) PNA lectin curve demonstrating the PD1-dependent sialic acid removal, shown as the emergence of asialoglycans (Galβ1-3GalNAc) revealed by removing α2-3 linked sialic acids on *O*-linked glycans.

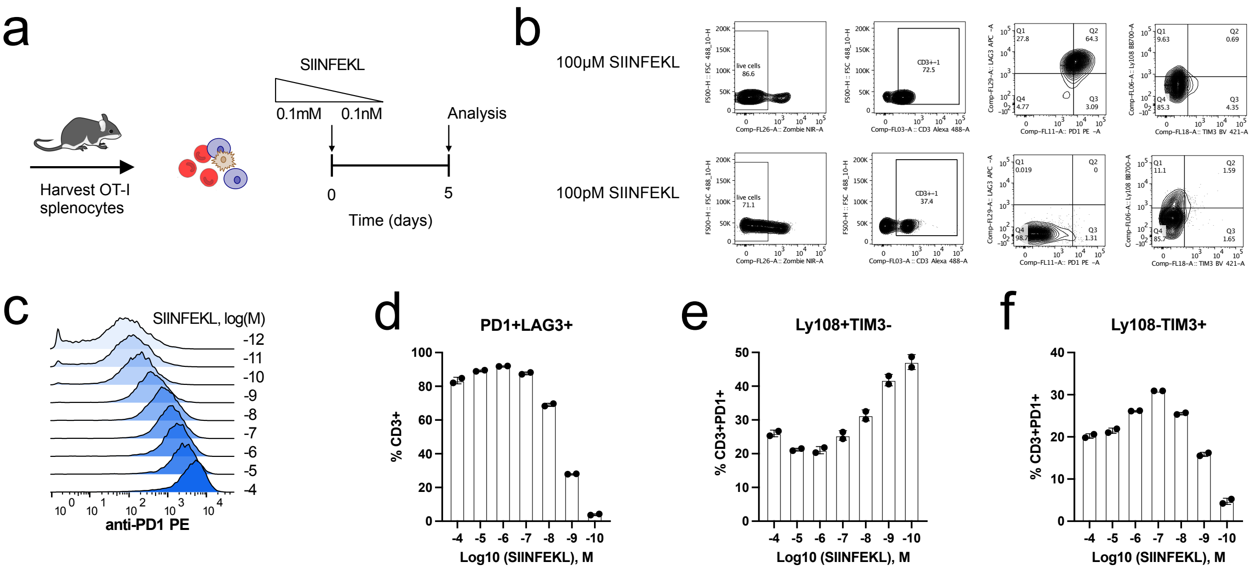

Figure S5. (**a**) Splenocytes from OT-I mice were stimulated with log10-fold dilutions of SIINFEKL for 5d and analyzed by flow cytometry for expression of T cell exhaustion markers PD1, LAG3, Ly108 and TIM3. (**b**) Representative gating strategy for 5d-stimualted OT-I cells. (**c**) PD1 expression depicted over a range of SIINFEKL concentrations. (**d**) OT-I effector phenotype as a function of stimulation strength with SIINFEKL peptide, indicating a local maximum in the expression of T cell exhaustion markers PD1 and LAG3 cells near 100nM. (**e**) The reciprocal loss of Ly108 expression, a surrogate marker of T cell stemness, indicates a terminally exhausted phenotype, further evidenced by elevated expression of TIM3 shown in (**f**).

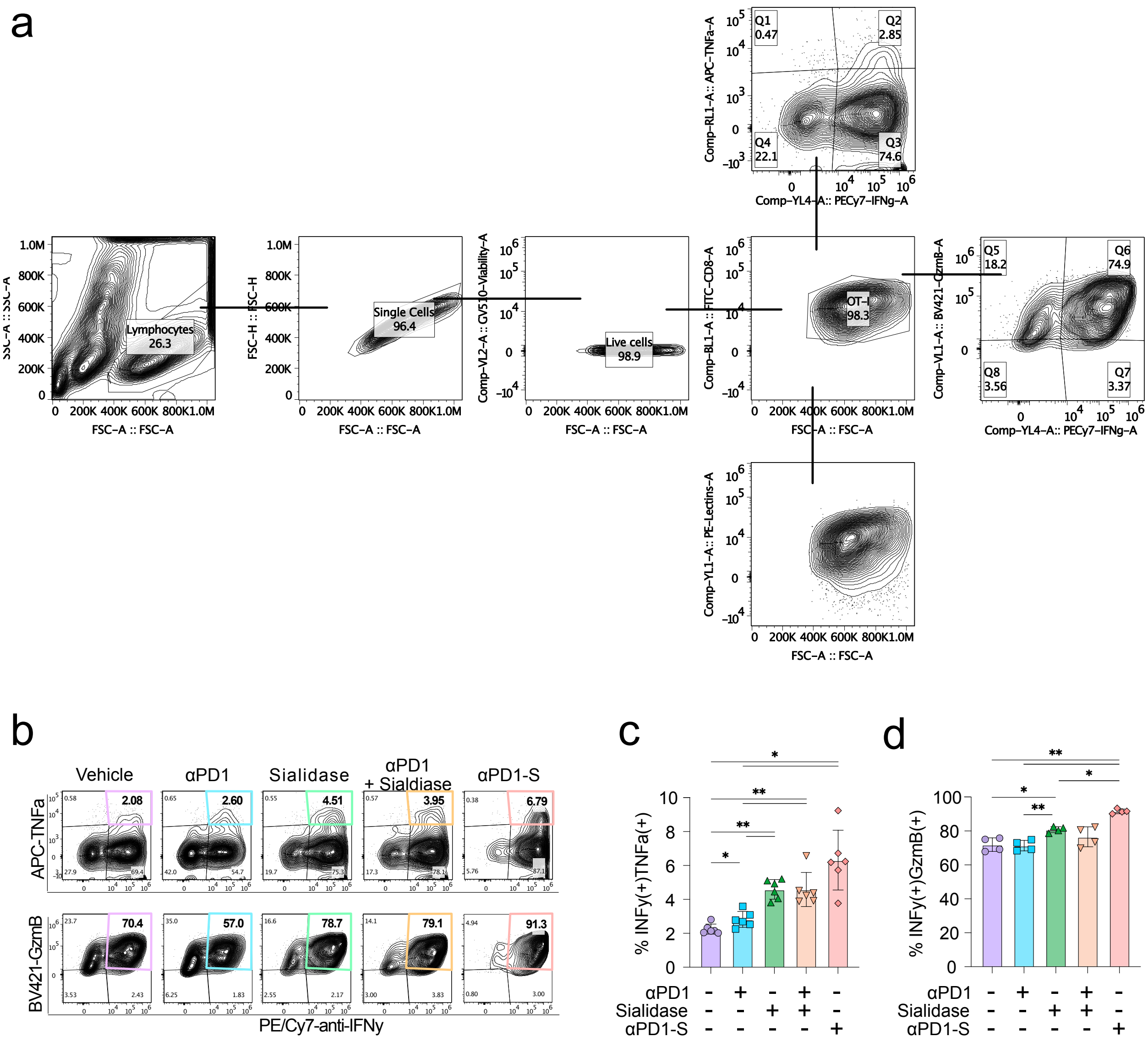

Figure S6. (**a**) Gating strategy for cell killing experiments described in Figure **4d-f** (**b**) Representative flow cytometry contour plots indicating cytokine levels (percent of total OT-I) following 24h of the indicated treatment (1nM final), quantified in (**c,d**).

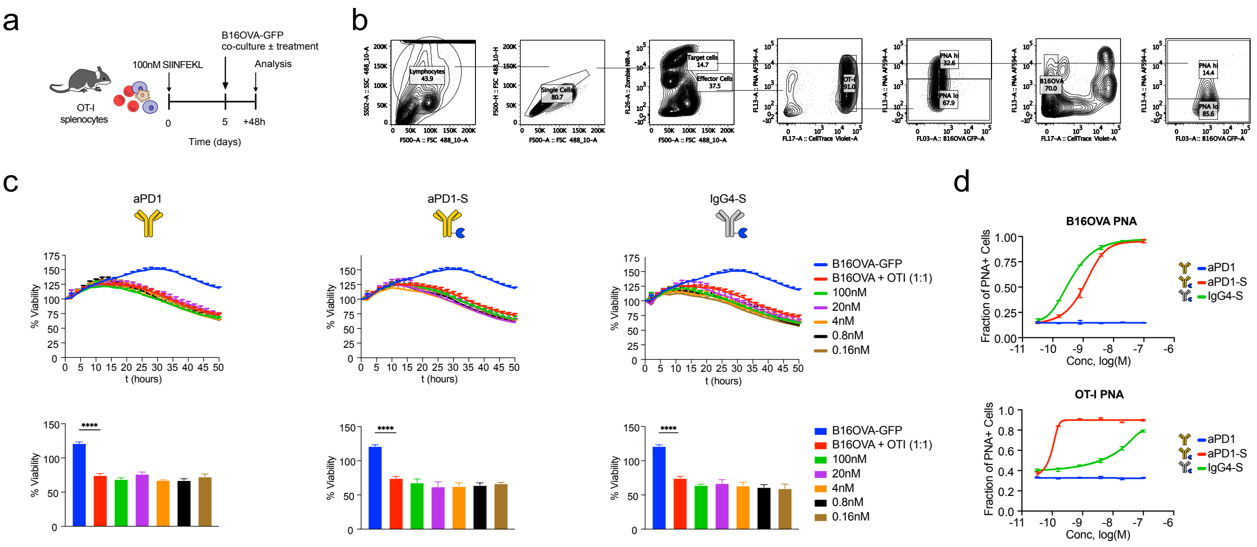

Figure S7. (**a**) Splenocytes from OT-I mice were stimulated 100nM SIINFEKL for 5d and subsequently CTV-stained and co-cultured with B16OVA-GFP cells at a ratio of 1:1 for 50h. (**b**) Representative gating strategy for analysis of co-cultured cells in **c**,**d**. (**c**) Incucyte growth curves indicating cell viability as determined by GFP fluorescence of B16OVA -GFP target cells over 48h. Cells were treated with titrations of anti-PD1 (top left), αPD1-S (top middle) or IgG4-S (top right). 48h endpoints are plotted for each treatment group (bottom). (**d**) Sialic acid analysis of OT-I and B16OVA-GFP cells following 1h of co-culture as determined by flow cytometry and PNA staining. Error bars represent standard deviation, p-values were determined by matched one-way ANOVA followed by Tukey's multiple comparisons test, where *$p < 0.05$, **$p \leq 0.01$, ***$p \leq 0.001$, ****$p \leq 0.0001$.

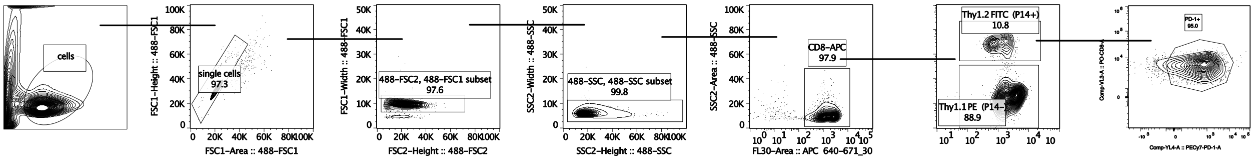

Figure S8. Gating strategy for P14/B16 cell killing experiments described in Figure **4i,j**

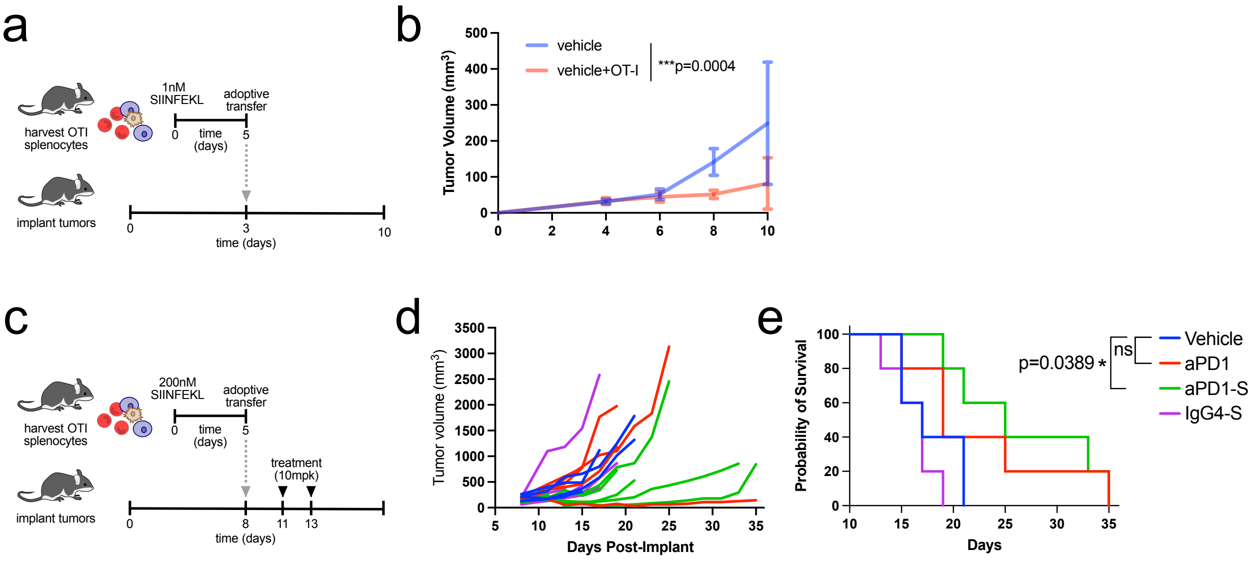

Figure S9. (**a**) OT-I splenocytes were stimulated for 5d *in vitro* using 1nM SIINFEKL and adoptively transferred into C57BL/6 mice bearing 3-day-old subcutaneous B16OVA tumors on their flank. (**b**) Averaged tumor volume measured over time (**c**) OT-I splenocytes were stimulated for 5d *in vitro* using 200nM SIINFEKL and adoptively transferred into C57BL/6 mice bearing 8-day-old subcutaneous B16OVA tumors on their flank. Mice were subsequently treated by intraperitoneal injection of vehicle or equimolar anti-PD1, aPD1-S*, or IgG4-S* on the indicated days for two total treatments. (**d**) Individual tumor volumes measured over time, with corresponding survival shown along a Kaplan-Meier plot in (**e**).

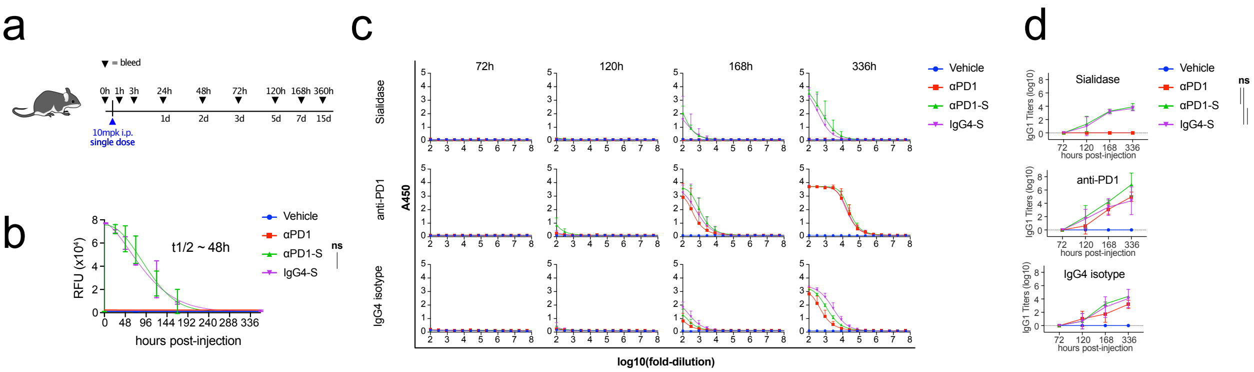

Figure S10. (**a**) Mice were treated by a single i.p. injection of vehicle, 1.33nmol anti-PD1 (10mg/kg), αPD1-S or IgG4-S. Serum was collected at the indicated time points and assayed for sialidase (4-MUNANA) activity in **b** or mouse IgG1 anti-drug antibodies (ADA's) in **c**,**d**. (**b**) 4-MUNANA activity of serum samples indicating no statistical differences in enzyme activity or serum half-life (t1/2~48h) between αPD1-S and IgG4-S. (**c**) ADA ELISAs indicating the emergence of sialidase, anti-PD1 or IgG4 isotype (motavizumab) anti-drug antibodies. Analytes indicated on the y-axis were separately plated and assayed for serum reactivity as determined using anti-mouse IgG1-HRP. (**d**) ADA titers, defined as the fold-dilution at which anti-mIgG1-HRP A450 measures twice the background, were plotted over time showing no statistical ADA differences between treatments. ns = not statistically significant.

a

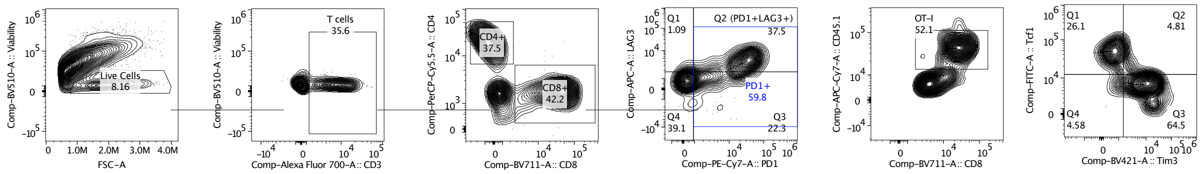

b

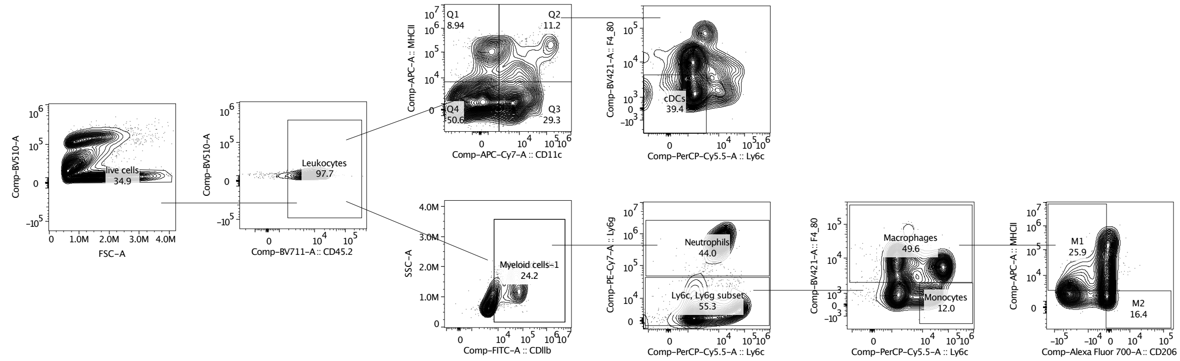

Figure S11. Representative gating strategies to analyze T cells (**a**) or myeloid cells (**b**) in tumor, spleen or tumor-draining lymph node.

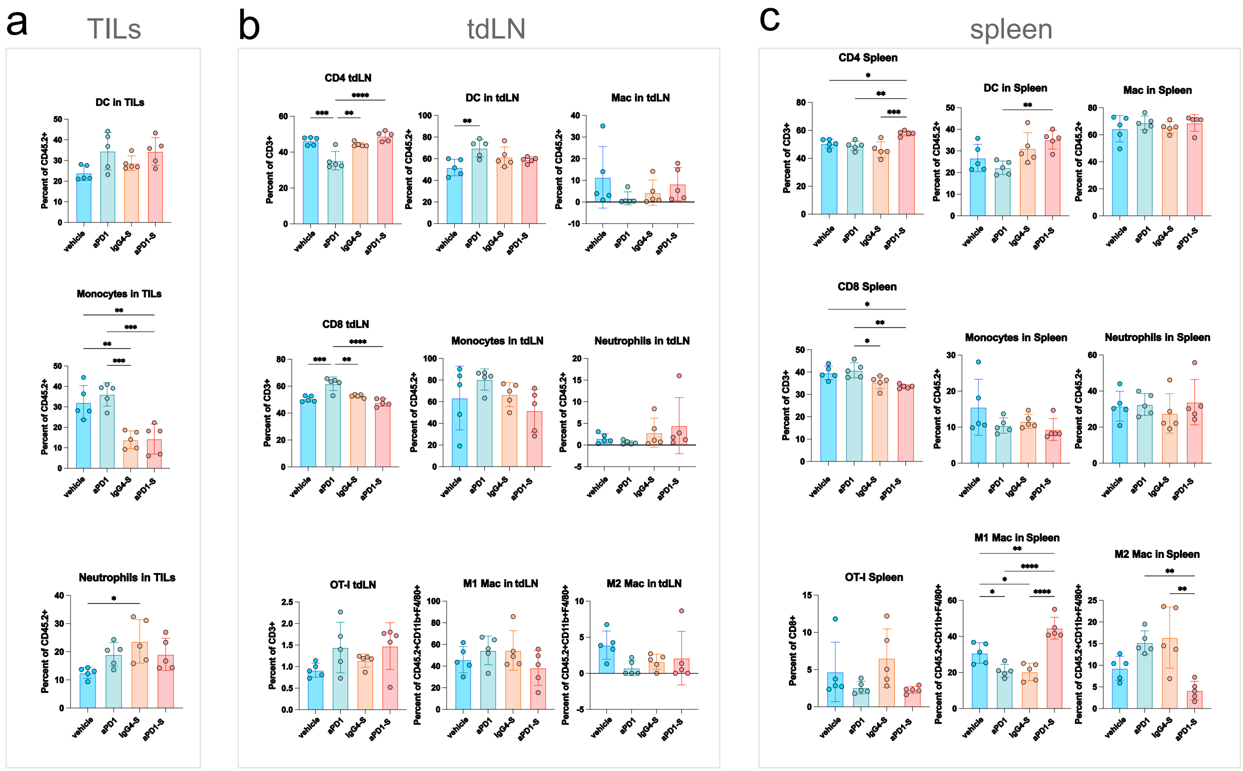

Figure S12 Frequencies of immune cell subsets determined in TILs in (**a**), tdLN in (**b**), or spleen in (**c**), represented as a percentage of the parent gate. Error represented as standard deviation *$p<0.05$; **p<0.01; ***p<0.001; ****p<0.0001 by 1-way ANOVA followed by Tukey's multiple comparisons test.

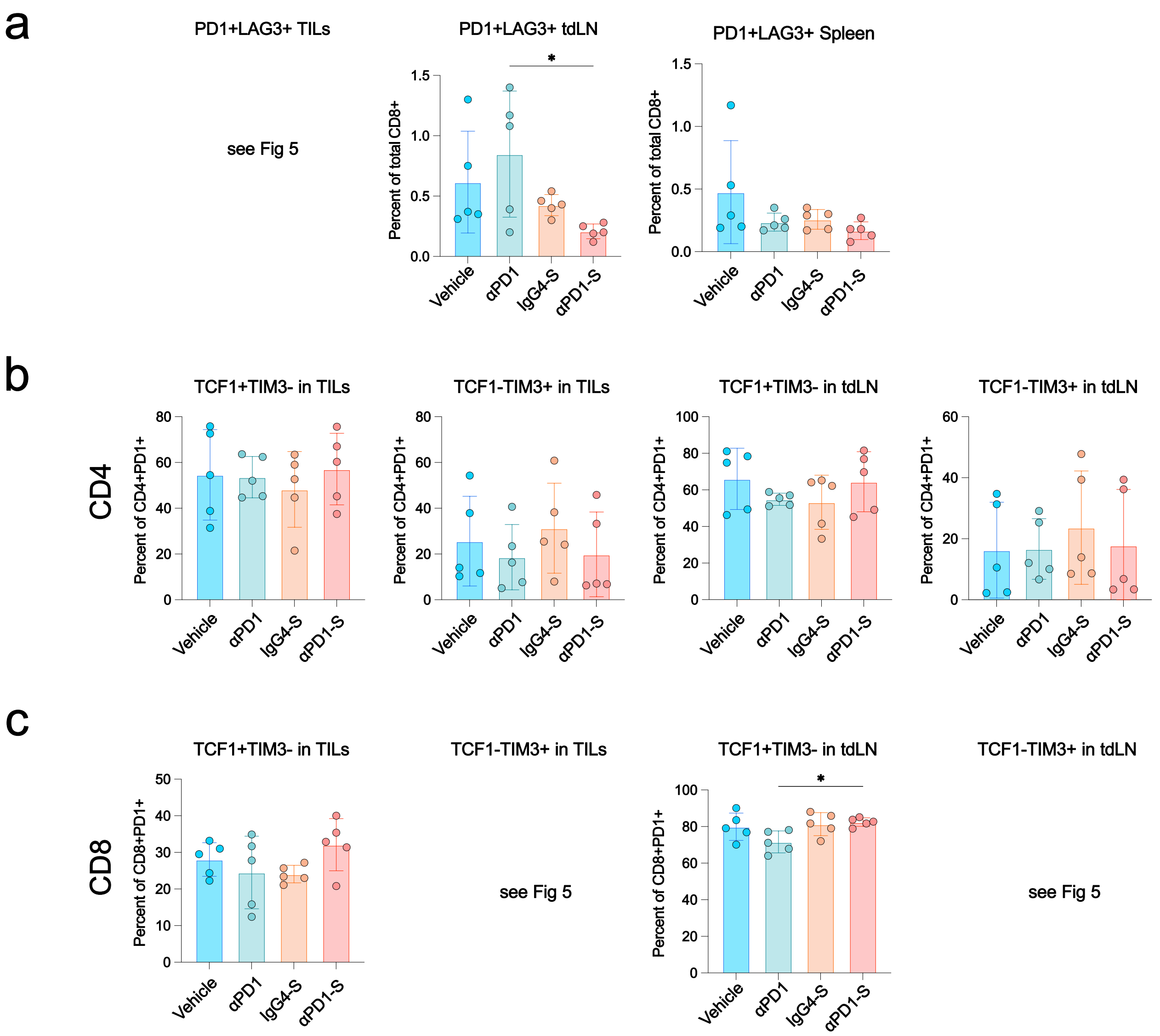

Figure S13 (**a**) Frequencies of PD1+LAG3+ cells in tdLN and spleen, expressed as a percent of total CD8+ cells. (**b**) Frequencies of TCF1±TIM3± cells expressed in TILs, or tdLN expressed as percent of CD4+PD1+ cells. (**c**) Frequencies of TCF1±TIM3± cells expressed in TILs, or tdLN expressed as percent of CD8+PD1+ cells. Error represented as standard deviation **p*<0.05; **p<0.01; ***p<0.001; ****p<0.0001 by 1-way ANOVA followed by Tukey's multiple comparisons test.

Table S1 – Fluorescently labeled antibodies used in this study

Supplier	Catalog #	Target / Specificity	Clone	Conjugate
BioLegend	109847	CD45.2	104	BV711
BioLegend	127617	Ly-6G	1A8	PE/Cy7
BioLegend	100434	CD4	GK1.5	PerCP/Cy5.5
BioLegend	100215	CD3	17A2	Alexa Fluor® 700
BioLegend	109109	CD279 (PD1)	RMP1-30	PE/Cy7
BioLegend	119723	CD366 (Tim-3)	RMT3-23	BV421
BioLegend	125209	CD223 (LAG-3)	C9B7W	APC
BioLegend	100740	CD8a	53-6.7	BV570
BD	564279	CD45	30-F11	BUV395
BD	562757	Siglec-F	E50-2440	PE-CF594
BD	565411	F4/80	T45-2342	BV421
BioLegend	101205	CD11b (mouse/human)	M1/70	FITC
BioLegend	128012	Ly-6C	HK1.4	PerCP/Cy5.5
thermofisher	48-0032-82	CD3	17A2	eFluor™ 450
BioLegend	405237	Streptavidin	N/A	Alexa Fluor® 647
vector labs	b-1075-5	Peanut Agglutinin (PNA)	N/A	Biotin
vector labs	b-1305-2	Sambucus Nigra Lectin (SNA)		Biotin
vector labs	b-1265-1	Maackia Amurensis Lectin II (MAL II)		Biotin
BioLegend	101320	CD16/32 (FcγIII/II) (TruStain FcX)	93	N/A (blocking reagent)

Table S2 – Amino acid sequences for proteins used in this study

Protein	AA sequence
SrtA(8M)-TEV-MBP-HIS	MQAKPQIPKDKSKVAGYIEIPDADIKEPVYPGPATREQLNRGVSF<br>AEENESLDDQNISIAGHTFIGRPNYQFTNLKAAKKGSMVYFKVG<br>NETRKYKMTSIRNVKPTAVGVLDEQKGKDKQLTLITCDDLNRET<br>GVWETRKIFVATEVKGGGGSGGGGSENLYFQGKIEEGKLVIWIN<br>GDKGYNGLAEVGKKFEKDTGIKVTVEHPDKLEEKFPQVAATGD<br>GPDIIFWAHDRFGGYAQSGLLAEITPDKAFQDKLYPFTWDAVRY<br>NGKLIAYPIAVEALSLIYNKDLLPNPPKTWEEIPALDKELKAKGKS<br>ALMFNLQEPYFTWPLIAADGGYAFKYENGKYDIKDVGVDNAGAK<br>AGLTFLVDLIKNKHMNADTDYSIAEAAFNKGETAMTINGPWAWS<br>NIDTSKVNYGVTVLPTFKGQPSKPFVGVLSAGINAASPNKELAKE<br>FLENYLLTDEGLEAVNKDKPLGAVALKSYEEELAKDPRIAATMEN<br>AQKGEIMPNIPQMSAFWYAVRTAVINAASGRQTVDEALKDAQTR<br>ITKHHHHHH
ST Sialidase	MGGGGGTVEKSVVFKAEGEHFTDQKGNTIVGSGSGGTTKYFRI<br>PAMCTTSKGTIVVFADARHNTASDQSFIDTAAARSTDGGKTWNK<br>KIAIYNDRVNSKLSRVMDPTCIVANIQGRETILVMVGKWNNNDKT<br>WGAYRDKAPDTDWDLVLYKSTDDGVTFSKVETNIHDIVTKNGTI<br>SAMLGGVGSGLQLNDGKLVFPVQMVRTKNITTVLNTSFIYSTDGI<br>TWSLPSGYCEGFGSENNIIEFNASLVNNIRNSGLRRSFETKDFG<br>KTWTEFPPMDKKVDNRNHGVQGSTITIPSGNKLVAAHSSAQNK<br>NNDYTRSDISLYAHNLYSGEVKLIDAFYPKVGNASGAGYSCLSY<br>RKNVDKETLYVVYEANGSIEFQDLSRHLPVIKSYNSLCTPSRGS<br>HHHHHH*
ST Sialidase R309A	MGGGGGTVEKSVVFKAEGEHFTDQKGNTIVGSGSGGTTKYFRI<br>PAMCTTSKGTIVVFADARHNTASDQSFIDTAAARSTDGGKTWNK<br>KIAIYNDRVNSKLSRVMDPTCIVANIQGRETILVMVGKWNNNDKT<br>WGAYRDKAPDTDWDLVLYKSTDDGVTFSKVETNIHDIVTKNGTI<br>SAMLGGVGSGLQLNDGKLVFPVQMVRTKNITTVLNTSFIYSTDGI<br>TWSLPSGYCEGFGSENNIIEFNASLVNNIRNSGLRRSFETKDFG<br>KTWTEFPPMDKKVDNRNHGVQGSTITIPSGNKLVAAHSSAQNK<br>NNDYTASDISLYAHNLYSGEVKLIDAFYPKVGNASGAGYSCLSY<br>RKNVDKETLYVVYEANGSIEFQDLSRHLPVIKSYNSLCTPSRGS<br>HHHHHH*
anti-mPD1	DMRVPAQLLGLLLLWLSGARCYELTQPPSASVNVGETVKITCSG<br>DQLPKYFADWFHQRSDQTILQVIYDDNKRPSGIPERISGSSSGT<br>TATLTIRDVRAEDEGDYYCFSGYVDSDSKLYVFGSGTQLTVLGG<br>PKSSPKVTVFPPSPEELRTNKATLVCLVNDFYPGSATVTWKANG<br>ATINDGVKTTKPSKQGQNYMTSSYLSLTADQWKSHNRVSCQVT<br>HEGETVEKSLSPAECLGGGGSEGRGSLLTCGDVEENPGPGSG<br>ATNFSLLKQAGDVEENPGPEFGLSWLFLVAILKGVQCEVRLLES<br>GGGLVKPEGSLKLSCVASGFTFSDYFMSWVRQAPGKGLEWVA<br>HIYTKSYNYATYYSGSVKGRFTISRDDSRSMVYLQMNNLRTEDT<br>ATYYCTRDGSGYPSLDFWGQGTQVTVSSASTKGPSVFPLAPCS

	RSTSESTAALGCLVKDYFPEPVTVSWNSGALTSGVHTFPAVLQS SGLYSLSSVVTVPSSSLGTKTYTCNVDHKPSNTKVDKRVESKYG PPCPPCPAPEFLGGPSVFLFPPKPKDTLMISRTPEVTCVVVDVS QEDPEVQFNWYVDGVEVHNAKTKPREEQFNSTYRVVSVLTVLH QDWLNGKEYKCKVSNKGLPSSIEKTISKAKGQPREPQVYTLPPS QEEMTKNQVSLTCLVKGFYPSDIAVEWESNGQPENNYKTTPPV LDSDGSFFLYSRLTVDKSRWQEGNVFSCSVMHEALHNHYTQKS LSLSLGLIKRENLYFQGGGGGSLPSTGEGGGGSSLCTPSRGS
IgG4 isotype (motavizumab)	MDMRVPAQLLGLLLLWLSGARCTGVHSDIQMTQSPSTLSASVG DRVTITCSASSRVGYMHWYQQKPGKAPKLLIYDTSKLASGVPSR FSGSGSGTEFTLTISSLQPDDFATYYCFQGSGYPFTFGGGTKVE IKRTVAAGPKSSPKVTVFPPSPEELRTNKATLVCLVNDFYPGSAT VTWKANGATINDGVKTTKPSKQGQNYMTSSYLSLTADQWKSHN RVSCQVTHEGETVEKSLSPAECLGGGGSEGRGSLLTCGDVEEN PGPGSGATNFSLLKQAGDVEENPGPEFGLSWLFLVAILKGVQCT GVHSQVQLQQPGAELVKPGASVKMSCKASGYTFTSYNMHWVK QTPGRGLEWIGAIYPGNGDTSYNQKFKGKATLTADKSSSTAYM QLSSLTSEDSAVYYCARSTYYGGDWYFNVWGAGTTVTVSAAST KGASTKGPSVFPLAPCSRSTSESTAALGCLVKDYFPEPVTVSW NSGALTSGVHTFPAVLQSSGLYSLSSVVTVPSSSLGTKTYTCNV DHKPSNTKVDKRVESKYGPPCPPCPAPEFLGGPSVFLFPPKSK DTLMISRTPEVTCVVVDVSQEDPEVQFNWYVDGVEVHNAKTKP REEQFNSTYRVVSVLTVLHQDWLNGKEYKCKVSNKGLPSSIEKT ISKAKGQPREPQVYTLPPSQEEMTKNQVSLTCLVKGFYPSDIAV EWESNGQPENNYKTTPPVLDSDGSFFLYSRLTVDKSRWQEGN VFSCSVMHEALHNHYTQKSLSLSLGLIKRENLYFQGGGGGSLPS TGEGGGGSSLCTPSRGS
anti-hPD1 (pembrolizumab)	MEFGLSWLFLVAILKGVQCQVQLVQSGVEVKKPGASVKVSCKA SGYTFTNYYMYWVRQAPGQGLEWMGGINPSNGGTNFNEKFKN RVTLTTDSSTTTAYMELKSLQFDDTAVYYCARRDYRFDMGFDY WGQGTTVTVSSASTKGPSVFPLAPCSRSTSESTAALGCLVKDY FPEPVTVSWNSGALTSGVHTFPAVLQSSGLYSLSSVVTVPSSSL GTKTYTCNVDHKPSNTKVDKRVESKYGPPCPPCPAPEFLGGPS VFLFPPKPKDTLMISRTPEVTCVVVDVSQEDPEVQFNWYVDGV EVHNAKTKPREEQFNSTYRVVSVLTVLHQDWLNGKEYKCKVSN KGLPSSIEKTISKAKGQPREPQVYTLPPSQEEMTKNQVSLTCLV KGFYPSDIAVEWESNGQPENNYKTTPPVLDSDGSFFLYSRLTVD KSRWQEGNVFSCSVMHEALHNHYTQKSLSLSLGLIKRENLYFQ GSSFLVQSGDGRLPSTGEGSLCTPSRGSGATNFSLLKQAGDVE ENPGPDMRVPAQLLGLLLLWLSGARCEIVLTQSPATLSLSPGER ATLSCRASKGVSTSGYSYLHWYQQKPGQAPRLLIYLASYLESGV PARFSGSGSGTDFTLTISSLEPEDFAVYYCQHSRDLPLTFGGGT KVEIKRTVAAPSVFIFPPSDEQLKSGTASVVCLLNNFYPREAKVQ WKVDNALQSGNSQESVTEQDSKDSTYSLSSTLTLSKADYEKHK VYACEVTHQGLSSPVTKSFNRGEC*

References

(1) Gray, M. A.; Stanczak, M. A.; Mantuano, N. R.; Xiao, H.; Pijnenborg, J. F. A.; Malaker, S. A.; Miller, C. L.; Weidenbacher, P. A.; Tanzo, J. T.; Ahn, G.; et al. Targeted glycan degradation potentiates the anticancer immune response in vivo. *Nat Chem Biol* **2020**. DOI: 10.1038/s41589-020-0622-x.
