# Supplementary material for: Targeting Sialidase to PD1 Enhances T cell Function and Tumor Control
Source: ACS Cent Sci. 2025 Jul 4;11(8):1417–27. doi: 10.1021/acscentsci.5c00510 (PMC12395300; doi:10.1021/acscentsci.5c00510)
Supplement: Supplementary file 3 [file oc5c00510_si_003.pdf]

oc-2025-00510s.R1

Name: Peer Review Information for "Targeting Sialidase to PD-1 Enhances T cell Function and Tumor Control"

First Round of Reviewer Comments

Reviewer: 1

Comments to the Author

In the present manuscript Garabedian et al explore the applicability of a sialidase-coupled anti PD-1 conjugate. They create both a WT and a sialidase mutant with reduced functional activity. They furthermore show that the aPD-1S reverses T cell exhaustion and augments tumor immune control.

This is a sound piece of work, significantly contributing to the arsenal of antibody-sialidase conjugates in pre-clinical evaluation. Although other sialidase-antibody constructs have been generated, these were mainly composed of sialidases coupled to tumor-targeting antibodies. To my knowledge this is the first sialidase-immune checkpoint inhibitor conjugate showing great promise in a pre-clinical tumor model. I have a few questions and remarks for the author to address:

1. I noticed that the authors have only used manual gating to analyze their flow cytometry data. Have the authors considered using unsupervised clustering (PARC/Flowsom) to address whether different immune cell subsets appear upon treatment with their different constructs?

2. In figure 3g: Do the authors have an explanation why the CD8 T cells appear more desialylated, even though PD-1 expression is higher on the CD4 T cells?

3. Interestingly, in figure 4d the authors show that the soluble sialidase increases (single) IFNy positive T cells to a greater extend than the aPD-1S (which does has the highest potency to enhance the IFNy/TNFa double positive T cells). Do the authors have an explanation for this?

4. Have the authors addressed viral titers in the LCMV model upon aPD-1S treatment?

5. I understand why the authors would opt to choose for the mutated sialidase in their tumor experiments. Yet, I wonder what the WT construct shows in terms of tumor growth reduction. I assume the authors tried this as well and in my opinion it would be of added value to add these data to the supplementary data.

6. For their tumor experiments the authors always employ an OT-I T cell transfer model, yet sialic acid mimetics are able to induce anti-tumor immunity in the B16-OVA model without the addition of OT-I T cells (e.g. Bull Cancer Research 2018). Have the authors employed their aPD-1S also in a model without additional OT-I T cells? What were the results there? I recommend to include these data as well. Why would the OT-I transfer be necessary if the anti-tumor immune response is already “strong” enough so that a sialic acid mimetic can overcome the tumor immune evasion?

7. Recent papers indicate that tumor-associated T cells acquire Siglec-9 acing as an additional checkpoint. When using the mutant sialidase-PD-1 construct, off target desialylation is limited, but in theory this would free up the Siglec-9 on the T cells (removing the cis-sialic acid interactions). In potential this T cell Siglec-9 could then engage sialic acids on the tumor and still dampen T cell activation. Do the authors have access to human tumor samples to culture tumor slices and evaluate their constructs and Siglec-9+ T cells under these settings?

Minor points:

The quality of the figures is not always the best and some of the data appears to be missing.

- The numbering of the supplemantry figures is odd, with S5 coming after S1 and before S2.

- Figure 3f: The indication of the glycans stained is only readable at very high magnification. Maybe add a legend explaining all the different colors to each fluorescent image?

- Figure 5e: The actual PNA staining indexes are missing from the figure.

- Figure S9b: The tumor growth curves are missing from the figure.

- Figure S10a: The writing above the timeline is unreadable

- Figure S12: What does TSTIM mean? I guess it is the sialidase conjugates, so I recommend to use the same wording throughout the whole manuscript, or please explain this in the figure legend.

Author's Response to Peer Review Comments:

10555 N. Torrey Pines Road, MB-212, La Jolla, CA 92037

May 24, 2025

ACS Central Science
Executive Editor
Re: Manuscript ID: oc-2025-00510s

Dear Editor,

Thank you for your consideration and review of our manuscript, entitled "**Targeting sialidase to PD-1 enhances T cell function and tumor control**" and for providing us the opportunity to submit a revised manuscript addressing the comments and concerns of the reviewers

In the revised manuscript and the attached responses to the reviewer's comments, we have made a concerted effort to address each concern. From the review we realized that the reviewer had received a poor-quality version of the figures that resulted from the conversion of our Word document to PDF format. We have taken precautions to make sure that this did not occur in this resubmission.

Please let us know if there is anything else we can provide to facilitate the review of this manuscript.

Thank you in advance for your consideration.

Sincerely,

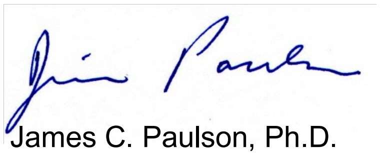

James C. Paulson, Ph.D.

Responses to Reviewer 1

Recommendation: Publish in ACS Central Science after minor revisions noted.

Comments:
In the present manuscript Garabedian et al explore the applicability of a sialidase-coupled anti PD-1 conjugate. They create both a WT and a sialidase mutant with reduced functional activity. They furthermore show that the aPD-1S reverses T cell exhaustion and augments tumor immune control.

This is a sound piece of work, significantly contributing to the arsenal of antibody-sialidase conjugates in pre-clinical evaluation. Although other sialidase-antibody constructs have been generated, these were mainly composed of sialidases coupled to tumor-targeting antibodies. To my knowledge, this is the first sialidase-immune checkpoint inhibitor conjugate showing great promise in a pre-clinical tumor model. I have a few questions and remarks for the author to address:

We thank the reviewer for the positive comments.

1. *I noticed that the authors have only used manual gating to analyze their flow cytometry data. Have the authors considered using unsupervised clustering (PARC/Flowsom) to address whether different immune cell subsets appear upon treatment with their different constructs?*

Neither we nor our collaborators at TSRI use PARC/Flowsom for analysis of flow cytometry data, and therefore, we may not appreciate the unique advantages it can offer for identifying unique immune cell subsets that would appear under different treatment conditions. However, we routinely use tSNE to identify immune cell subsets and manually assign identities to clusters by evaluating their expression of specific markers included in our panel (e.g., Figure 3g). We now understand that PARC/Flowsom offers an independent way of identifying subsets with unsupervised clustering, and will apply it in future studies where comprehensive immune profiling is central to our research objectives.

2. *In figure 3g: Do the authors have an explanation why the CD8 T cells appear more desialylated, even though PD-1 expression is higher on the CD4 T cells?*

Indeed, it seems counter intuitive that CD4 T cells express higher levels of PD-1 than CD8 T cells, yet following treatment with our αPD1-Sialidase exhibit lower binding of PNA used as a measure of desialylation. However, it is known that activated CD8 T cells become more PNA positive than activated CD4 T cells as a result of the reduced activity of the sialyltransferase ST3Gal I, and the de novo production of CD45 with under sialylated O-linked glycans (Amado et al., 2004). Thus, CD8 T cells may intrinsically have higher levels of glycoproteins with Oglycans (e.g. CD45) that become PNA positive upon treatment with sialidase. Transcriptomic- and glyco-profiling of activated T cells have also revealed more pronounced reduction of another sialyltransferase, ST6Gal I in CD8 vs CD4 T cells. (Figure 5F, Izzati et al., 2024). For these reasons we do not expect PNA staining (or staining with other lectins) to precisely correlate with the extent of PD1 expression. We thank the reviewer for this observation and have included a brief discussion in the Results addressing this point.

3. *Interestingly, in figure 4d the authors show that the soluble sialidase increases (single) IFNy positive T cells to a greater extend than the aPD-1S (which does has the highest potency to enhance the IFNy/TNFa double positive T cells). Do the authors have an explanation for this?*

We thank the reviewer for highlighting the fact that in Figure 4d, the top panel, the total INFγ positive cells in the top panel (analyzing double positive INFγ/TNFa cells) were higher in the sialidase-treated cells than those in the αPD1-S treated cells. However, this was not the case in the lower panel, which analyzed INFγ/Granzyme B double-positive cells. Since the %INFγ cells should be the same for the same cells, we realized that we had selected representative panels for the αPD1-S cells from two different data sets.

The experiment involved activating cells treated with either 1 nM sialidase or 0.1 nM sialidase, with six replicates of each. There was variation in the extent of T cell activation using %INFγ as a reference. We have now replaced Figure 4d with

representative data from one of the 0.1 nM data sets, and bar graphs in Figure 4d and 4f that combine the data from all 6 replicates. The representative panels in 4d were chosen as a complete data set from one replicate that was representative of the average activation in each condition as represented in 4f. We also provided representative flow diagrams and bar graph data for all the experiments in the 1 nM data sets in Figure S6.

4. Have the authors addressed viral titers in the LCMV model upon aPD-1S treatment?

We only performed *in* vitro experiments using the spleens from LCMV-infected animals using virus and protocols of the laboratory of Dr. John Tejaro, who is the TSRI expert in LCMV acute and chronic infections. The spleens were used as a source of antigen-specific PD-1 expressing ‘exhausted’ T cells for the *in vitro* experiments. Although we have not done in vivo experiments with αPD-1-S in the LCMV model, we acknowledge that demonstrating viral clearance *in vivo* could provide insights into the therapeutic potential of αPD-1–S in chronic viral infection.

5.I understand why the authors would opt to choose for the mutated sialidase in their tumor experiments. Yet, I wonder what the WT construct shows in terms of tumor growth reduction. I assume the authors tried this as well and in my opinion it would be of added value to add these data to the supplementary data.

We initially evaluated a variety of models, including MC38, CT26, and B16F10. In early experiments, we tested peritoneal administration of αPD1 and αPD1-S to suppress tumor growth, and neither showed a significant reduction in tumor growth. This led us to test the B16OVA model with OT-I cells to improve the probability of demonstrating the enhanced immune response in antigen specific T cells. By that time, we had concluded that the wildtype sialidase would give significant off-target desialylation, as observed by Grey et al. 2020, so we elected to use the sialidase with reduced activity. We agree that it would be informative to know if the WT sialidase would provide a superior (or worse) outcome, but we have not done the experiment in the described B16OVA model. As further discussed below, we have now mentioned our preliminary experiments testing these tumor models in the discussion.

6. For their tumor experiments the authors always employ an OT-I T cell transfer model, yet sialic acid mimetics are able to induce anti-tumor immunity in the B16-OVA model without the addition of OT-I T cells (e.g. Bull Cancer Research 2018). Have the authors employed their aPD1S also in a model without additional OT-I T cells? What were the results there? I recommend to include these data as well. Why would the OT-I transfer be necessary if the anti-tumor immune response is already “strong” enough so that a sialic acid mimetic can overcome the tumor immune evasion?

As discussed above, we did initially test both αPD1 and αPD1-S in several tumor models, finding that they were resistant to control by both agents. For this reason we focused on the B16OVA model with adoptive transfer of OT-I cells to control the level of antigen-specific T cells. As we report, in this model there was significant improvement in tumor control by αPD1, which was further enhanced by αPD1-S.

While Bull et al. did indeed see that treatment of B16OVA tumors by intra-tumoral injection of the sialyltransferase inhibitor $3F_{ax}$-NeuAc had strong tumor control without OT I cells. The fact that 'cured mice' rejected tumor cells upon rechallenge (Bull et al. Fig 1G) and that the antitumor response was CD8 T cell-dependent strongly suggests that the innate immune system has the capability to exert antigen-specific tumor control if sufficiently stimulated. However, the mechanism by which intratumor injection of this sialyltransferase inhibitor, in particular, if it was mediated by 'desialylation' of tumor cells or immune cells or both, has not been established.

Notably, Bull et al. also saw reduced tumor growth in B16-F10 wild type tumors, and we saw nothing with our treatments with αPD1 or αPD1-S with the wild type sialidase. We have now added this information to the discussion since, as the referee pointed out, it is relevant to optimizing strategies targeting the glyco-immune axis for the treatment of cancer.

7. Recent papers indicate that tumor-associated T cells acquire Siglec-9 acing as an additional checkpoint. When using the mutant sialidase-PD-1 construct, off target desialylation is limited, but in theory this would free up the Siglec-9 on the T cells (removing the cis-sialic acid interactions). In potential this T cell Siglec-9 could then engage sialic acids on the tumor and still dampen T cell activation. Do the authors have access to human tumor samples to culture tumor slices and evaluate their constructs and Siglec-9+ T cells under these settings?

We agree that this would be a highly relevant direction for next steps. Although we have a few connections to the health community there is no clear path to obtain appropriate samples at present. It will take time to establish the relationships and models necessary to test the suggested approach.

Minor points:

The quality of the figures is not always the best and some of the data appears to be missing.

We downloaded the MS from the journal to see what the reviewer saw. Unfortunately, we discovered that the figures were corrupted upon conversion from our Word document to a PDF, either on our end during submission or in the conversion to a PDF by the journal. As a result, the quality of the figures was highly compromised in many respects, including loss of colors in the bars of the graphs. Our apologies for not catching the conversion error. In our revised submission, we have provided corrected files and have ensured that the quality is not lost on conversion to PDF. All relevant points regarding figure organization and content have been addressed and clarified in this revision.

- *The numbering of the supplementary figures is odd, with S5 coming after S1 and before S2.*

Thank you for catching this error. We have renumbered Supplementary figures according to their appearance in the manuscript.

- *Figure 3f: The indication of the glycans stained is only readable at very high magnification. Maybe add a legend explaining all the different colors to each fluorescent image?*

- *Figure 5e: The actual PNA staining indexes are missing from the figure*

- *Figure S9b: The tumor growth curves are missing from the figure.*

- *Figure S10a: The writing above the timeline is unreadable*

We thank the reviewer for identifying these issues related to figure readability and completeness. Upon review, we found that they were all attributable the errors with the PDF file conversion discussed above. We have checked each point and believe that they have been resolved.

- *Figure S12: What does TSTIM mean? I guess it is the sialidase conjugates, so I recommend to use the same wording throughout the whole manuscript, or please explain this in the figure legend.*

We thank the reviewer for pointing out this oversight. "TSTIM" is our previous designation for the construct now referred to as "αPD1-S" throughout the manuscript. We have made this change and searched the manuscript to make sure there are no other occurrences of TSTIM.

oc-2025-00510s.R2

Name: Peer Review Information for "Targeting Sialidase to PD-1 Enhances T cell Function and Tumor Control"

Second Round of Reviewer Comments

Reviewer: 1

Comments to the Author

I thank the authors for their revisions and their replies to my comments.

Author's Response to Peer Review Comments:

Dear Editor,

We were pleased to receive the provisional acceptance of our manuscript, entitled "Targeting

sialidase to PD-1 enhances T cell function and tumor control". We have addressed the minor

formatting items requested for the supporting information paragraph in the main manuscript and added

the ‘title page’ information on each supporting information file.

We hope that our changes meet the formatting needs of ACS Central Science; if not, we will be happy

to make any additional revisions.

Thank you for your efficient handling of our manuscript.
